# One‐Step Aqueous Synthesis of Glycosyl Pyridinium Salts, Electrochemical Study, and Assessment of Utility as Precursors of Glycosyl Radicals Using Photoredox Catalysis

**DOI:** 10.1002/open.202500183

**Published:** 2025-04-21

**Authors:** Daniel Chong, Paula A. Brooksby, Antony J. Fairbanks

**Affiliations:** ^1^ School of Physical and Chemical Sciences University of Canterbury Private Bag 4800 Christchurch 8140 New Zealand

**Keywords:** carbohydrates, cyclic voltammetry, glycosyl, photoredox, protecting group free, pyridiniums

## Abstract

A single step method for the production of unprotected glycosyl pyridinium salts has been developed involving treatment of the unprotected sugar with a pyridine, triethylamine, and either 2‐chloro‐1,3‐dimethylimidazolinium chloride (DMC) or 2‐chloro‐1,3‐dimethyl‐1 H‐benzimidazol‐3‐ium chloride (CDMBI) as an activator, in aqueous solution. Reaction efficiency is sensitive to steric effects, and in particular, *ortho*‐substitution of the pyridine ring significantly decreased conversion to product; *para*‐substitution of the pyridine ring is well tolerated. Cyclic voltammetry reveals that glycosyl pyridinium salts possess reduction potentials in the range of −0.9 to −1.4 V versus the standard calomel electrode, which are modulated by the electron effects of ring substituents. However, glycosyl pyridiniums are not found to be useful precursors for the production of glycosyl radicals under photoredox conditions.

## Graphical abstract

Unprotected glycosyl pyridinium salts may be accessed in a single step from the corresponding unprotected sugar in aqueous solution. Whilst meta‐ and para‐substitution is well tolerated, ortho substitution of the N‐heterocycle inhibits product formation. Electrochemical studies revealed reduction potentials in the range of −0.9 to −1.4 V. Attempted generation and trapping of glycosyl radicals by photoredox methods was unsuccessful.

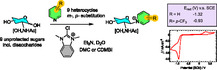

## 1 Introduction

Glycosyl pyridinium salts were first synthesized in 1910 by Fischer and Raske.^[^ ^1^ ^]^ It was however only many years later that interest in the study of these compounds resurfaced. Firstly, seminal work performed by Lemieux^[^ ^2^, ^3^ ^]^ into their structure gave rise to the concept of the reverse anomeric effect, due to the preference for such compounds to adopt a conformation in which the pyridinium ring adopted an equatorial rather than axial orientation. Although the validity of the “reverse anomeric effect” has subsequently been debated,^[^ ^4^ ^]^ subsequently, a considerable number of unprotected glycosyl pyridinium and related salts were synthesized and examined as substrates for glycosidases, principally by Sinnott and coworkers^[^ ^5^, ^6^, ^7^, ^8^, ^9^, ^10^, ^11^ ^]^. Recently, the photolysis of glycosyl pyridiniums was investigated,^[^ ^12^ ^]^ and some of their structures were determined by crystallography.^[^ ^13^ ^]^

As part of an ongoing program into the development of protecting group free transformations of unprotected sugars, we became interested in the synthesis of unprotected glycosyl pyridinium salts and related compounds. In particular, given the widespread utility of 2,4,6‐triphenyl pyridinium species, commonly known as Katritzky salts,^[^ ^14^ ^]^ for the generation of radicals^[^ ^15^, ^16^, ^17^, ^18^ ^]^ under photoredox conditions,^[^ ^19^, ^20^, ^21^ ^]^ the production of glycosyl pyridinium salts and investigation of their redox properties appeared to be a worthwhile and novel line of investigation. Application of photoredox chemistry to unprotected carbohydrates is an appealing prospect, as photoredox chemistry is compatible with aqueous reaction conditions.^[^ ^22^ ^]^ Numerous researchers have already realized the potential advantages of the generation of glycosyl radicals under photoredox conditions.^[^ ^23^ ^]^ Correspondingly, multiple anomeric derivatives, such as glycosyl halides,^[^ ^24^, ^25^, ^26^ ^]^ sulfoxides,^[^ ^27^, ^28^ ^]^ sulfinates,^[^ ^29^, ^30^ ^]^ sulfones,^[^ ^31^, ^32^, ^33^ ^]^ and dihydropyridyl esters,^[^ ^34^, ^35^, ^36^, ^37^, ^38^ ^]^ amongst others^[^ ^39^ ^]^ have been developed as photoredox activatable precursors of glycosyl radicals. However, in the vast majority of cases, reactions either involved photoredox activation of protected carbohydrate derivatives, or in the few cases where unprotected glycosyl radicals were formed, protecting group manipulations were required in order to make the glycosyl radical precursors themselves. Indeed, only very recently were methods for the generation of glycosyl radicals reported that were completely devoid of any protecting group manipulations.^[^ ^37^, ^38^, ^40^, ^41^ ^]^

It was considered that given the widespread utility of pyridinium salts as radical precursors^[^ ^42^, ^43^, ^44^ ^]^ following facile one‐electron reduction, access to glycosyl pyridiniums and investigation of their redox properties may provide a new entry into carbohydrate derivatives, which may in turn be converted into glycosyl radicals under mild reaction conditions.

## 2 Synthesis of Glycosyl Pyridiniums and Related Compounds

Although a single report exists on the preparation of unprotected glycosyl pyridiniums by reaction of glycosyl amines with 1‐(2,4‐dinitrophenyl)pyridinium chloride,^[^ ^45^ ^]^ the vast majority of previous synthetic routes to glycosyl pyridinium salts have all involved multiple steps and protecting group manipulations. Typically, the peracetylated glycosyl pyridinium salt is formed by the reaction of a fully protected glycosyl halide with a pyridine or related heterocycle, and the sugar protecting groups are then removed by acid‐catalyzed ester hydrolysis. It was considered, however, that glycosyl pyridiniums and related compounds may be accessible in a single step from unprotected reducing sugars by use of the extremely useful aqueous‐stable dehydrating agent 2‐chloro‐1,3‐dimethylimidazolinium chloride (DMC **1a**, Shoda's reagent).^[^ ^46^ ^]^ DMC‐mediated reactions have proven widely applicable for the one‐step derivatization of unprotected carbohydrates in aqueous solution, allowing the production of a range of glycosides, including glycosyl azides and triazoles,^[^ ^47^, ^48^, ^49^ ^]^ thioglycosides,^[^ ^50^, ^51^, ^52^ ^]^ and related compounds,^[^ ^40^, ^53^, ^54^, ^55^, ^56^ ^]^ and even *O*‐glycosides^[^ ^57^, ^58^ ^]^ and disaccharides^[^ ^59^ ^]^ without recourse to any protecting group manipulations.

Studies began with an investigation of the production of glycosyl pyridinium salts using glucose **2a** as the substrate (**Table** **1**). The reaction of glucose with 3 equivalents of pyridine, Et_3_N as a base, and DMC **1a** as an activator in D_2_O at 0 °C for 1 h gave the desired glycosyl pyridinium **2b** in a modest 30% yield, as only the β‐anomer (Table 1, entry 1); **2b** was isolated as the carbonate salt after purification by ion exchange chromatography eluting with 1M aqueous ammonium carbonate. The complete 1,2‐*trans*‐sterocontrol of this reaction is consistent with numerous previous observations of DMC‐mediated nucleophilic substitution at the anomeric center of unprotected sugars and the intermediacy of a 1,2‐anhydro sugar intermediate.^[^ ^60^ ^]^ Increasing the amount of Et_3_N added to 9 equivalents increased the conversion to product (62%, entry 2), as did the use of increased equivalents of pyridine (entries 3 and 4, 80, and 86% respectively). The use of Hünig's base instead of Et_3_N reduced the conversion to **2b** (44%, entry 5), whereas the omission of added base (entry 6) did not produce any reaction, indicating that pyridine alone is unsuccessful in effecting activation of sugars by DMC, even when used in large excess. The use of water as reaction solvent instead of D_2_O resulted in a slightly less effective reaction (65%, entry 7) in line with previous observations of the beneficial use of D_2_O as reaction solvent, presumably due to decreased rates of hydrolysis arising from a solvent kinetic isotope effect.^[^ ^61^, ^62^ ^]^ While the use of a mixed solvent system (entry 8, D_2_O/MeCN, 1:1) had a negligible effect on the reaction outcome, variation of reagent concentration did produce slight variations in the yield of **2b**; conversion to product was more efficient at the higher reaction concentration tested (entry 10), while slightly less efficient at the lower concertation (entry 9), in line with an interplay between intermolecular processes competing with solvent hydrolysis of activated intermediates. Application of optimized conditions was then applied to the production of significant quantities of **2b,** but purification of the reaction mixture proved difficult and resulted in considerably lower yields of isolated product (entries 2 and 3, c.f. isolated product yields and yields calculated by NMR analysis). In particular, we encountered a persistent reaction impurity that resulted from a side reaction of DMC, which coeluted with the product during cation exchange chromatography. The modified activator 2‐chloro‐1,3‐dimethyl‐1 H‐benzimidazol‐3‐ium chloride (CDMBI **1b**),^[^ ^63^ ^]^ developed by Shoda as an alternative to DMC with more favorable properties to aid product purification, was therefore investigated, in an attempt to facilitate product isolation. The use of CDMBI **1b** as an activator proved equally efficient to the use of DMC **1a** (entries 11 and 12), and product purification was found to be considerably easier as CDMBI‐related side products did not coelute with the product. CDMBI **1b** was therefore selected as the activator to be used in subsequent experiments, and the use of 9 equivalents of both pyridine and Et_3_N as bases was deemed optimal (Table 1 entry 12).

**Table 1:** Optimization of the production of glycosyl pyridinium salt **2b** from glucose **2a**.

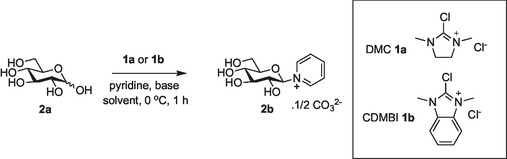						
Entry	Solvent	Concentration/[mM]	Pyridine [equiv.]	Base [equiv.]	Activator [equiv.]	Yield/%a)
1	D_2_O	280	3	Et_3_N (3)	DMC **1a** (3)	30
2	D_2_O	280	3	Et_3_N (9)	DMC **1a** (3)	62 [42]b)
3	D_2_O	280	6	Et_3_N (9)	DMC **1a** (3)	80 [57]b)
4	D_2_O	280	9	Et_3_N (9)	DMC **1a** (3)	86
5	D_2_O	280	6	iPr_2_NEt (9)	DMC **1a** (3)	44
6	D_2_O	280	15	–	DMC **1a** (3)	0
7	H_2_O	280	6	Et_3_N (9)	DMC **1a** (3)	65
8	D_2_O:MeCN 1:1	280	6	Et_3_N (9)	DMC **1a** (3)	79
9	D_2_O	93	6	Et_3_N (9)	DMC **1a** (3)	63
10	D_2_O	830	6	Et_3_N (9)	DMC **1a** (3)	85
11	D_2_O	280	6	Et_3_N (9)	CDMBI **1b** (3)	70 [54]b)
12	D_2_O	280	9	Et_3_N (9)	CDMBI **1b** (3)	76 [58]b)

a) Determined by 1 H NMR by comparing the integrals of the anomeric proton of the product and that of unprotected sugar.

b) Isolated yield of purified product.

**Scheme 1:**
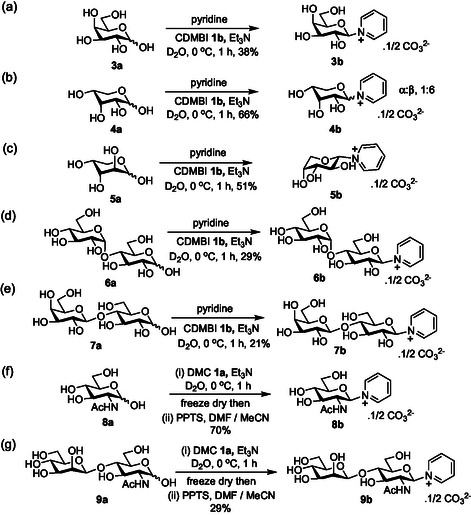
Application to other sugar substrates.

Next, these optimized conditions were applied to several different sugar substrates in order to demonstrate reaction scope. In the case of 2‐hydroxy sugars, monosaccharides galactose **3a**, and arabinose **5a** reaction proceeded smoothly to provide the corresponding 1,2‐*tran*s pyridinium salts (**Scheme** **1**a,c). Notably, the arabino product **5b** adopts a ^1^C_4_ conformation in which the pyridine adopts an equatorial orientation in line with the reverse anomeric effect. In the case of ribose **4a**, the reaction was not completely stereoselective and a minor amount of the 1,2‐*cis* isomer was also formed (Scheme 1b, α:β, 1:6). The reaction was equally applicable to disaccharides; maltose **6a** and lactose **7a** yielded the corresponding pyridinium salts **6b** and **7b** respectively (Scheme 1d,e) albeit in reduced yields due to competitive formation of their 1,6‐anhydro derivatives.

However, in the case of 2‐acetamido sugars, such as GlcNAc **8a**, the attempted reaction under these conditions gave predominantly the corresponding glycosyl oxazoline, and only low amounts of the desired pyridinium species were formed (≈10%). However, the desired pyridinium salt could readily be formed by first deliberately forming the glycosyl oxazoline using DMC and Et_3_N in D_2_O. Next, following the removal of the solvent by freeze‐drying the crude reaction mixture and the dissolution of the residue in a MeCN/DMF mixed solvent system, the addition of pyridinium *para*‐toluenesulfonate (PPTS) induced the opening of the oxazoline ring to give the desired β‐pyridinium salt **8b** (Scheme 1f). Further application was demonstrated by the conversion of the Manβ(1→4)GlcNAc disaccharide **9a** into the corresponding disaccharide pyridinium salt **9b** in a 29% isolated yield (Scheme 1g). Thus, a one‐pot two‐step procedure via an intermediate oxazoline which is not isolated or purified readily gives access to pyridinium salts of 2‐acetamido sugars.

The next application of the optimized reactions conditions was examined using a variety of different substituted pyridines and a small selection of other heterocycles possessing a pyridine‐like nitrogen (**Table** **2**). In each case again, purification by ion exchange chromatography, involving elution with 1M aqueous ammonium carbonate, meant that isolated compounds were produced as the carbonate salts. A 70% conversion of glucose into the corresponding pyridinium salt **2c** was observed when nicotinamide **10c** was used in place of pyridine (entry 1). Isolation of **2c** was hindered by the large excess of nicotinamide present, as this was not removed by ion exchange chromatography. However, when 2‐methyl pyridine **10d** was used as a nucleophile, only a very low conversion to the corresponding pyridinium **2d** was observed (23%, entry 2) indicating the detrimental steric effect of the *ortho* methyl group on the reaction outcome. Furthermore, when 2,4,6‐collidine **10e** was used as a substrate, no formation of the desired product **2e** was observed (entry 3). However, substitution of the 4‐position of pyridine was well tolerated, and the 4‐substituted derivatives **10f‐h** were all converted into the corresponding pyridiniums **2f‐h** with acceptable conversions (entries 4–6). It should be noted that in each of these cases, a mixed solvent system (D_2_O:MeCN, 1:1) was used to ensure solubility of the pyridine derivative. Several of the substituted pyridinium salts were found to be quite unstable. *p*‐Methoxy substituted pyridinium salt **2f** was found to be reasonably unstable and underwent facile hydrolysis. Trifluoromethyl pyridinium derivative **2h** was particularly unstable under the mildly basic conditions used for purification by ion exchange, and so acetic acid was added to neutralize carbonate immediately after purification; thus trifluoromethylpyridinium derivative **2h** was isolated as the acetate (rather than carbonate) salt. Interestingly, in the case of 4‐cyanopyridine **10i**, the product isolated (53%, entry 7) was identified as the pyridone **4i,** which was presumably formed by facile hydrolysis of the expected 4‐cyanopyrinidum product via an addition/elimination mechanism (S_N_‐Ar/AE).

**Table 2:** Application of optimized reaction conditions to substituted pyridines and other heterocycles.

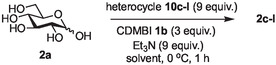				
Entry	Heterocycle	Solvent	Product	Yield/%a)
1	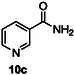	D_2_O	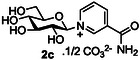	70
2	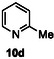	D_2_O	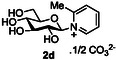	23 [18]b)
3	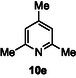	D_2_O	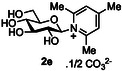	0
4	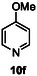	D_2_O:MeCN 1:1	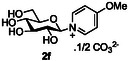	73
5	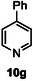	D_2_O:MeCN 1:1	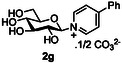	62
6	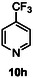	D_2_O:MeCN 1:1	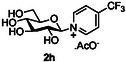	50 [17]b)
7	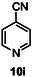	D_2_O:MeCN 1:1	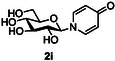	57 [53]b)
8	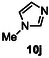	D_2_O	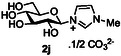	75 [60]b)
9	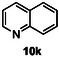	D_2_O	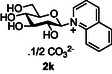	<5
10	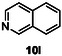	D_2_O	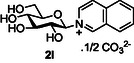	64

a) Determined by 1 H NMR by comparing the integrals of the anomeric proton of the product and that of unprotected sugar.

b) Isolated yield of purified product.

Next, the applicability to other heterocycles containing a “pyridine‐like” nitrogen was examined. The use of N‐methyl imidazole **10j** gave the corresponding imidazolinium salt **2j** in good conversion (75%, entry 8). However, the attempted reaction using quinoline **10k** only produced the corresponding quinolinium species **2k** in very low conversion (<5% conversion observed by NMR, entry 9), again highlighting the detrimental effect of substitution *ortho* to the pyridine nitrogen. Correspondingly, the use of isoquinoline **10l** was more successful, and good conversion of glucose to the corresponding isoquinolinium salt **2l** (64%, entry 10) was observed.

## 3 Electrochemical Studies

**Figure 1:**
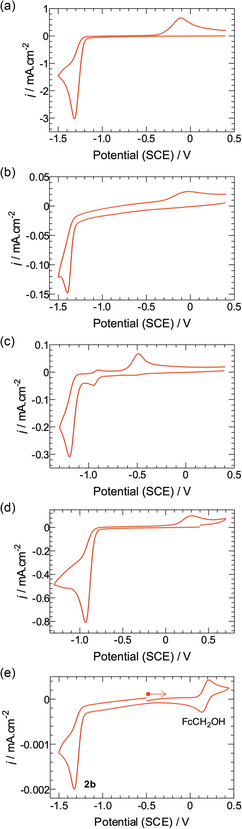
Cyclic voltammograms (scan rate = 100 mV.s^−1^) in 0.1 M Na_2_SO_4_ aqueous electrolyte (10 mL, N_2_ sparged) solution. a) **2b** (54 mg); b) **2d** (2.5 mg); c) **2g** (22 mg); d) **2h** (22 mg). e) Cyclic voltammogram of an aqueous 0.1 M KCl electrolyte solution containing 8.28 mM **2b **+ 1.66 mM hydroxymethyl ferrocene (FcCH_2_OH). The voltammogram was started at −0.5 V and scanned toward positive potentials initially. The molar ratio of **2b**:FcCH_2_OH was 4.99:1, and the average peak current ratio for **2b**:FcCH_2_OH was 5.14:1.

With a demonstrated one‐step route to glycosyl pyridiniums in hand, attention turned to the examination of their redox properties prior to potential application for radical generation under photoredox or other reductive conditions. Reduction potentials of all compounds investigated are provided in **Table** **3**, and cyclic voltammograms are shown in **Figure** **1**. For analytes, scan rates were varied between 20 mV s^−1^ and 2 V s^−1^ (see Supporting Information, Figure S1–S4).

**Table 3:** Redox potentials of glycosyl pyridinium species.

Entry	Substrate	*E*_red_ [V]vs. SCE	*E*_ox_ [V] vs. SCE	Reversibility
1	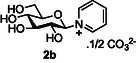	−1.32	−0.11	Irreversible
2	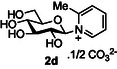	−1.40	−0.04	Irreversible
3	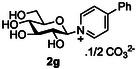	−1.20	−0.48	Irreversible
4	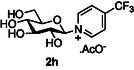	−0.93	+0.30	Irreversible

CV studies indicated that the reduction potentials of the glycosyl pyridinium salts were modulated by substituents on the pyridine ring, in line with expected substituent effects. Thus, while the *E*_red_ of the unsubstituted pyridinium salt **2b** was measured as −1.32 V (Figure 1a), substitution of the ring with an electron‐donating methyl substituent increased this to −1.40 V for compound **2d** (Figure 1b). However, modification by the addition of the phenyl group reduced the observed reduction potential relative to that of the unsubstituted pyridine ring to −1.20 V for compound **2g** (Figure1c), presumably as a result of lowering the energy of the LUMO by increasing conjugation. Furthermore, the introduction of the strongly electron‐withdrawing CF_3_ substituent in compound **2h** decreased the observed *E*_red_ to −0.93 V (Figure 1d).

Next, to determine the number of electrons transferred in the reduction of **2b**, a CV study was performed (Figure 1e) on a mixture comprising a known amount of **2b** and a known amount of a well‐defined redox species; the water‐soluble strictly outer sphere 1 electron‐transfer redox species hydroxymethyl ferrocene (FcCH_2_OH) was used for the comparison. Based on the assumption that the diffusion coefficients of **2b** and FcCH_2_OH are relatively similar, then if the peak current ratios were found to be the same as the molar ratios, it could be concluded that the reduction of **2b** was a 1‐electron‐transfer event.^[^ ^64^ ^]^ Alternately, if the peak current ratios were found to be double the molar ratios, then the reduction of **2b** must involve 2 electrons. In this instance, a solution containing a concentration ratio of 4.99:1 mM of **2b**:FcCH_2_OH in aqueous 0.1 M KCl solution was examined (Figure 1e). The current ratios were measured using both the anodic (4.35:1) and cathodic (5.92:1) peak currents, with the average being 5.14:1, that is, the same as the molar ratios of the two analytes. It was therefore concluded that the reduction of **2b** is a single electron transfer event.

## 4 Photoredox Studies

Given the demonstrated electrochemical one‐electron reduction of these glycosyl pyridinium species, as a next step, the glucosyl pyridinium salt **2b** (*E*_red_ − 1.32 V) and trifluoromethylpyridinium salt **2h** (*E*_red_ − 0.93 V) were investigated as potential precursors of glycosyl radicals under photoredox conditions. Typically, procedures attempted involved irradiation of the substrate in a range of solvents with blue light (455 nm LEDs) at room temperature for 24 h, in the presence of the photoredox catalyst and excess Et_3_N as a stoichiometric reductant. Reactions were performed in the presence of electron‐deficient alkene acceptors (either methyl acrylate or acrylonitrile) to intercept any glycosyl radicals formed via Giese‐type reaction. For both salts **2b** and **2h** catalysts investigated included transition metal‐based species, such as [Ru(bpy)_3_](PF_6_)_2_ and [Ir(dFCF_3_ppy)_2_(dtbbpy)](PF_6_), and organic photoredox catalysts, such as 9‐mesityl‐10‐methylacridinium perchlorate (Mes‐Acr‐ClO_4_) and 1,2,3,5‐tetrakis(carbazol‐9‐yl)‐4,6‐dicyanobenzene (4CzIPN). However, the only carbohydrate products isolated from any of these reactions were either recovered starting material, glucose, or 1,6‐anyhdro glucose.

**Scheme 2:**
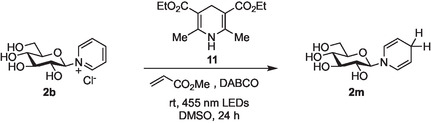
Production of glycosyl dihydropyridines via attempted photoredox generation of glycosyl radicals from glycosyl pyridiniums.

Next, the use of Hanztsch ester **11** was investigated as a potential photoactivator of salt **2b**, again in the presence of methyl acrylate in order to intercept any glycosyl radicals formed, and a base, typically DABCO. In these cases, all of the starting material **2b** was consumed, but the corresponding glucosyl dihydropyridine **2m** was identified as the main reaction product by NMR (**Scheme** **2**).

Initially, we suspected that the formation of **2m** in the presence of Hantzsch ester **11** indicated that a second one‐electron reduction of a putative radical intermediate occurred faster than homolytic cleavage of the C—N bond^[^ ^17^ ^]^ to produce the desired glycosyl radical. Indeed, slow homolytic cleavage of C—N bonds has previously been identified as a limiting factor in the application of pyridinium species as a source of alkyl radicals; typically, steric acceleration of bond cleavage can be achieved by the introduction of substituents at the 2 and 6 positions on the pyridine ring, for example, as in Katritzky salts, and steric effects generally outweigh electronic effects arising from pyridine ring substitution.^[^ ^17^ ^]^ However, in our case, subsequent investigations revealed that **2m** was also formed under similar conditions in the absence of light. In this latter case, reduction most probably occurs via hydride transfer from Hanztsch ester **11**.

In the presence of light, whether the formation of **2m** occurs either by two sequential one‐electron reductions, or by hydride transfer, is difficult to dissect. The CV studies performed on **2b** (Figure 1e) indicated that under electrochemical conditions, the reduction of the pyridinium ring does indeed occur by a one electron process. Whilst it is tempting to extrapolate this finding and assume that reduction under photoredox conditions may also occur via an initial one‐electron reduction, it is clear that under these conditions no glycosyl radical was formed using any of the photocatalytic systems investigated. Unfortunately, the propensity of unprotected glycosyl pyridinium salts to decompose precluded variation of reaction conditions, for example, the use of elevated temperatures, in order to try and favor the generation of glycosyl radicals. Consequently, the development of glycosyl pyridiniums as readily accessible precursors to unprotected glycosyl radicals remains an as‐yet‐unrealized challenge.

## 5 Conclusions

A one‐step aqueous method for the production of glycosyl pyridinium salts has been developed which allows access to a range of compounds in a single operation using unprotected sugars. The reaction is applicable to both mono‐ and disaccharides of 2‐hydroxy sugars. For 2‐acetamido sugars, a slightly modified two‐step procedure via intermediate oxazoline formation and then opening under acidic conditions also gives glycosyl pyridinium salts. The reaction efficiency is, however, sensitive to steric effects, and, in particular, *ortho*‐substitution of the pyridine ring significantly decreases conversion to the glycosyl pyridinium product. Studies by cyclic voltammetry indicate that glycosyl pyridinium salts possess reduction potentials in the range of −0.9 to −1.4 V versus saturated calomel electrode (SCE) and that these potentials are modulated by substituents on the pyridine ring. However, despite the fact that these reduction potentials are well within the range of several common photoredox catalytic systems, the glycosyl pyridiniums examined were not found to be effective precursors of glycosyl radicals under photoredox conditions.

## 6 Experimental Section

6.1

6.1.1

##### General

Reactions conducted at 0 °C were cooled by means of an ice bath. Solvent was removed under reduced pressure using a Buchi^TM^ rotary evaporator. Reagents were used as supplied without further purification unless otherwise stated. Thin‐layer chromatography (t.l.c.) was carried out on Merck Silica Gel 60F_254_ aluminum‐backed plates. Visualization of the plates was achieved using a UV lamp (lmax = 254 or 365 nm) and/or ammonium molybdate (5% in 2M H_2_SO_4_) and/or 5% H_2_SO_4_ in ethanol. Flash column chromatography was carried out using Sorbsil C60 40/60 silica. Melting points were recorded on an Electrothermal melting point apparatus. Proton and carbon nuclear magnetic resonance (*δ*_H_, *δ*_C_) spectra were recorded on JEOL ECZ400S and JEOL ECZ600R spectrometers. All chemical shifts are quoted on the δ‐scale in ppm using residual solvent as an internal standard. ^1^ H and ^13^C spectra were assigned using COSY, DEPT, HSQC, HMQC, or HMBC. High‐resolution mass spectra were recorded on a Bruker maXis 3 G UHR‐TOF mass spectrometer using electrospray ionization or chemical ionization techniques as stated. M/z values are reported in Daltons. Optical rotations were measured on a Rudolph Research Analytical AUTOPOL IV automatic polarimeter with a Ceramic Quartz cell with a path length of 50 mm and an inner diameter of 5 mm and are quoted in units of °.cm^2^ g^−1^.

##### Cyclic Voltammetry

All voltammetry was performed in a conventional single‐compartment 3‐electrode glass cell using an Eco Chemie Autolab PGSTAT302 potentiostat, operating NOVA software version 2.1. The working electrode was a glassy carbon disc (GC, 3 mm diameter) sealed in Teflon. The auxiliary electrode was a large area gold wire, and the reference electrode was a KCl SCE. The voltammograms were collected at 100 mV s^−1^ (unless otherwise stated) and at room temperature. The GC electrode was polished using an alumina slurry (50 nm particle size) on a microcloth, followed by rinsing with copious amounts of water, then sonication in a water bath to remove any remaining alumina, and finally dried with N_2_. The electrochemical cell was sparged with N_2_ to remove oxygen from the solution, and an N_2_ atmosphere was maintained above the solution during measurements. All electrochemistry was performed in either a 0.1 M Na_2_SO_4_ or 0.1 M KCl aqueous electrolyte solution containing a known amount of the electroactive species. All solutions were made using ultrapure water, with 18.2 MOhm.cm resistance. The current densities are quoted with respect to the geometric area of the working electrode, with the roughness factor of the GC electrode being close to 2. No IR corrections were applied to the measurements.

##### Synthetic Methods: General Procedure A: Production of Glycosyl Pyridinium Salts Using 2‐Hydroxy Sugars

The sugar (1 equiv.) and triethylamine (9 equiv.) were dissolved in D_2_O or D_2_O:MeCN (1:1) (typically 2 mL). The pyridine derivative (9 equiv.) was added, and the mixture was stirred at 0 °C for 10 min. CDMBI **1b** (3 equiv.) was added, and the mixture was stirred at 0 °C for 1 h. After this time, the reaction mixture was diluted with water, washed with ethyl acetate, and coevaporated with 35% w/w aqueous ammonium hydroxide (typically 2 × 8.5 mL). The residue was charged onto a cation exchange resin (Amberlite IR120 (H^+^), dry weight ≈15 g per 100 mg of starting sugar, previously washed with 1 M aqueous ammonium carbonate) and washed with water. The product was eluted from the resin with 1 M aqueous ammonium carbonate, concentrated in vacuo at 40 °C, and lyophilized to give the corresponding glycosyl pyridinium salt.

##### Synthetic Methods: General Procedure B: Production of Glycosyl Pyridinium Salts Using 2‐Acetamido Sugars

The 2‐acetamido sugar (1 equiv.) and triethylamine (9 equiv.) were dissolved in water, and the mixture was cooled to 0 °C. DMC **1a** (3 equiv.) was then added, and the reaction mixture was stirred at 0 °C for 30 min. After this time, t.l.c. (typically chloroform to methanol, 2:1) indicated complete consumption of starting material and formation of a less polar major product. The reaction mixture was diluted with water and lyophilized. The residue was suspended in a 10:1 (v/v) mixture of dry acetonitrile and dry dimethylformamide. The mixture was sonicated for 1 min; then, powdered 4 Å molecular sieves were added, and the mixture was stirred at rt under N_2_ for 30 min. Pyridinium *p*‐toluenesulfonate (6 equiv.) was added, and the reaction mixture was stirred at rt under N_2_ for 1 h. After this time, t.l.c. (typically, chloroform to methanol, 2:1) indicated complete consumption of starting material and formation of a major product close to the baseline (e.g., R_f_ 0.05). The reaction mixture was diluted with methanol, filtered through a pad of Celite, and the filtrate was concentrated in vacuo. The residue was dissolved in 35% w/w aqueous ammonium hydroxide, and the mixture was then concentrated in vacuo. This operation was then repeated. The residue was then dissolved in water and charged onto a cation exchange resin (Amberlite IR120 (H^+^), dry weight ≈15 g per 100 mg of starting sugar, previously washed with 1 M aqueous ammonium carbonate) and washed with water. The pyridinium salt was then eluted with 1 M aqueous ammonium carbonate, concentrated in vacuo at 40 °C, and lyophilized to give the corresponding glycosyl pyridinium salt.

##### Compound Data: N‐(β‐D‐Glucopyranosyl) Pyridinium Carbonate **2b**

General procedure A using D‐glucose **2a** (100 mg, 0.56 mmol), triethylamine (0.69 mL, 5.00 mmol), D_2_O (2 mL), pyridine (0.40 mL, 5.00 mmol), and CDMBI **1b** (362 mg, 1.67 mmol) gave compound **2b** (89 mg, 59%) as a white solid. [α]_D_^20^ = +47 (*c* = 1.0 in water); IR (neat, cm^−1^): *ν*_max_ = 3062 (w) (C—H), 1627 (s) (C=C), 1019 (vs.) (C—O); ^1^ H NMR (400 MHz, D_2_O, 25 °C): *δ* = 9.07 (d, ^3^ *J*(H,H) = 6.1 Hz, 2 H; 2 × Ar‐H), 8.73 (t, ^3^ *J*(H,H) = 7.8 Hz, 1 H; Ar‐H), 8.21 (t, ^3^ *J*(H,H) = 7.1 Hz, 2 H; 2 × Ar‐H), 5.81 (d, ^3^ *J*(H,H) = 8.8 Hz, 1 H; H‐1), 4.02 (dd, ^2^ *J*(H,H) = 12.2 Hz, ^3^ *J*(H,H) = 1.5 Hz, 1 H; H‐6’), 3.93–3.84 (m, 2 H; H‐5, H‐6), 3.80 (at, ^3^ *J*(H,H) = 9.2 Hz, 1 H; H‐3), 3.71 (at, ^3^ *J*(H,H) = 9.2 Hz, 1 H; H‐4), 3.67 (at, ^3^ *J*(H,H) = 9.0 Hz, 1 H; H‐2); ^13^C NMR (100 MHz, D_2_O, 25 °C): *δ* = 161.9 (s, CO_3_), 148.3 (d, Ar‐C), 142.1 (d, 2 × Ar‐C), 128.2 (d, 2 × Ar‐C), 95.1 (d, C‐1), 79.5 (d, C‐5), 75.4 (d, C‐3), 74.1 (d, C‐2), 68.7 (d, C‐4), 60.4 (t, C‐6); HRMS‐ES: calcd for C_11_H_16_NO_5_ [*M*]^+^: 242.1023, found: 242.1029.

##### Compound Data: 1‐(β‐D‐Glucopyranosyl)‐2‐Methylpyridinium Carbonate **2d**

General procedure A using D‐glucose **2a** (100 mg, 0.56 mmol), triethylamine (0.69 mL, 5.00 mmol), D_2_O (2 mL), 2‐methylpyridine **10d** (0.49 mL, 5.00 mmol), and CDMBI **1b** (362 mg, 1.67 mmol) gave compound **2d** (29 mg, 18%) as a pink solid. [α]_D_^20^ = +54 (*c* = 1.0 in water); IR (neat, cm^−1^): *ν*_max_ = 3195 (w) (C—H), 1628 (vs.) (C=C), 1063 (vs.) (C—O); ^1^H NMR (400 MHz, D_2_O, 25 °C): *δ* = 9.07 (d, ^3^ *J*(H,H) = 6.5 Hz, 1 H; Ar‐H), 8.51 (t, ^3^ *J*(H,H) = 7.8 Hz, 1 H; Ar‐H), 8.03 (t, ^3^ *J*(H,H) = 7.1 Hz, 1 H; Ar‐H), 8.00 (d, ^3^ *J*(H,H) = 8.0 Hz, 1 H; Ar‐H), 6.14 (d, ^3^ *J*(H,H) = 8.8 Hz, 1 H; H‐1), 3.98 (ad, ^2^ *J*(H,H) = 10.9 Hz, 1 H; H‐6’), 3.91–3.83 (m, 3 H, H‐3; H‐5, H‐6), 3.81 (at, ^3^ *J*(H,H) = 8.8 Hz, 1 H; H‐2), 3.71 (at, ^3^ *J*(H,H) = 9.2 Hz, 1 H; H‐4), 2.99 (s, <3 H [D_2_O exchange]; CH_3_); ^13^C NMR (100 MHz, D_2_O, 25 °C): *δ* = 147.1 (d, Ar‐C), 141.3 (d, Ar‐C), 130.0 (d, Ar‐C), 126.2 (d, Ar‐C), 89.6 (d, C‐1), 79.5 (d, C‐5), 75.4 (d, C‐3), 74.9 (d, C‐2), 68.9 (d, C‐4), 60.5 (t, C‐6); HRMS‐ES: calcd for C_12_H_18_NO_5_ [*M*]^+^: 256.1179, found: 256.1184.

##### Compound Data: 1‐(β‐D‐Glucopyranosyl)‐4‐Phenylpyridinium Carbonate **2g**

General procedure A using D‐glucose **2a** (100 mg, 0.56 mmol), triethylamine (0.69 mL, 5.00 mmol), 4‐phenylpyridine **10g** (775 mg, 5.00 mmol), and CDMBI **1b** (362 mg, 1.67 mmol) gave compound **2g** (31 mg, 16%) as a yellow solid. [α]_D_^20^ = +10 (*c* = 0.4 in water); IR (neat, cm^−1^): *ν*_max_ = 3303 (br) (O—H), 1636 (s) (C=C), 1015 (vs.) (C—O); ^1^H NMR (400 MHz, D_2_O, 25 °C): *δ* = 9.02 (d, ^3^ *J*(H,H) = 6.1 Hz, 2 H; 2 × NC*H*CH), 8.43 (d, ^3^ *J*(H,H) = 6.9 Hz, 2 H; 2 × NCHC*H*), 8.01 (dd, ^3^ *J*(H,H) = 7.9, 1.8 Hz, 2 H; 2 × Ar‐H), 7.81–7.58 (m, 3 H; 3 × Ar‐H), 5.79 (d, ^3^ *J*(H,H) = 8.6 Hz, 1 H; H‐1), 4.03 (dd, ^2^ *J*(H,H) = 12.3 Hz, ^3^ *J*(H,H) = 1.9 Hz, 1 H; H‐6’), 3.93 (dd, ^2^ *J*(H,H) = 12.4 Hz, ^3^ *J*(H,H) = 5.3 Hz, 1 H; H‐6), 3.87 (ddd, ^3^ *J*(H,H) = 9.7 Hz, 5.2 Hz, 1.8 Hz, 1 H; H‐5), 3.84–3.78 (m, 1 H; H‐3), 3.76–3.69 (m, 2 H; H‐2, H‐4); ^13^C NMR (100 MHz, D_2_O, 25 °C): *δ* = 161.6 (s, CO_3_), 141.9 (d, 2 × Ar‐C), 133.7 (s, Ar‐C), 132.7 (d, Ar‐C), 132.0 (s, Ar‐C), 129.8 (d, 2 × Ar‐C), 128.3 (d, 2 × Ar‐C), 124.9 (d, 2 × Ar‐C), 94.5 (d, C‐1), 79.5 (d, C‐5), 75.5 (d, C‐3), 74.0 (d, C‐2), 68.8 (d, C‐4), 60.5 (t, C‐6); HRMS‐ES: calcd for C_17_H_20_NO_5_ [*M*]^+^: 318.1136, found: 318.1132.

##### Compound Data: 4‐(Trifluoromethyl)‐1‐(β‐D‐Glucopyranosyl) Pyridinium Acetate **2h**

General procedure A was followed using D‐glucose **2a** (400 mg, 2.22 mmol), triethylamine (2.78 mL, 20.0 mmol), D_2_O/MeCN (1:1, 8 mL), 4‐(trifluoromethyl)pyridine **10 h** (2.31 mL, 20.0 mmol), and CDMBI **1b** (1.45 g, 6.67 mmol). Following purification by ion exchange chromatography, the resulting solution was acidified to pH ≈6 by the dropwise addition of glacial acetic acid, concentrated in vacuo at 40 °C, and then lyophilized repeatedly to give compound **2h** (141 mg, 17%) as an orange solid. [α]_D_^20^ = +40 (*c* = 1.0 in water); IR (neat, cm^−1^): *ν*_max_ = 3130 (w) (C—H), 1556 (s) (CO_2_^−^), 1150 (vs.) (C‐F), 1104 (vs.) (C‐F), 1019 (vs.) (C—O); ^1^ H NMR (400 MHz, D_2_O, 25 °C): *δ* = 9.40 (d, ^3^ *J*(H,H) = 6.5 Hz, 2 H; 2 × NC*H*CH), 8.60 (d, ^3^ *J*(H,H) = 5.5 Hz, 2 H; 2 × NCHC*H*), 5.96 (d, ^3^ *J*(H,H) = 8.9 Hz, 1 H; H‐1), 4.03 (ad, ^2^ *J*(H,H) = 11.1 Hz, 1 H; H‐6’), 3.96–3.86 (m, 2 H; H‐5, H‐6), 3.82 (at, ^3^ *J*(H,H) = 9.2 Hz, 1 H; H‐3), 3.73 (at, ^3^ *J*(H,H) = 9.2 Hz, 1H; H‐4), 3.65 (at, ^3^ *J*(H,H) = 9.0 Hz, 1 H; H‐2); ^19^ F NMR (376 MHz, D_2_O, 25 °C): *δ* = –65.4 (s, 3 F; CF_3_); ^13^C NMR (100 MHz, D_2_O, 25 °C): *δ* = 181.5 (s, *C*(O)CH_3_), 147.2 (q, ^2^ *J*(C‐F) = 36.4 Hz, *C*CF_3_), 144.2 (d, 2 × N*C*HCH), 125.2 (q, ^4^ *J*(C‐F) = 3.6 Hz, 2 × NCH*C*H), 120.9 (q, ^1^ *J*(C‐F) = 274.5 Hz, CF_3_), 95.5 (d, C‐1), 79.7 (d, C‐5), 75.4 (d, C‐3), 74.2 (d, C‐2), 68.6 (d, C‐4), 60.4 (t, C‐6), 23.3 (q, C(O)*C*H_3_); HRMS‐ES: calcd for C_12_H_15_F_3_NO_5_ [*M*]^+^: 310.0897, found: 310.0891.

##### Compound Data: 1‐(β‐D‐Glucopyranosyl)‐4(1 H)‐Pyridone **2i**

General procedure A using D‐glucose **2a** (100 mg, 0.56 mmol), triethylamine (0.69 mL, 5.00 mmol), D_2_O/MeCN (1:1, 2 mL), 4‐cyanopyridine **10i** (520 mg, 5.00 mmol), and CDMBI **1b** (362 mg, 1.67 mmol) gave compound **2i** (75 mg, 53%) as a yellow solid. [α]_D_^20^ = +23 (*c* = 1.0 in water); IR (neat, cm^−1^): *ν*_max_ = 3041 (w) (C—H), 1636 (s) (C=C), 1015 (vs.) (C—O); ^1^H NMR (400 MHz, D_2_O, 25 °C): *δ* = 8.05 (d, ^3^ *J*(H,H) = 7.7 Hz, 2 H; 2 × NC*H*CH), 6.64 (d, ^3^ *J*(H,H) = 7.7 Hz, 2 H; 2 × NCHC*H*), 5.31–5.16 (m, 1 H; H‐1), 3.95 (dd, ^2^ *J*(H,H) = 12.6 Hz, 3* J*(H,H) = 2.2 Hz, 1 H; H‐6’), 3.83 (dd, ^2^ *J*(H,H) = 12.6 Hz, ^3^ *J*(H,H) = 5.3 Hz, 1 H; H‐6), 3.74–3.66 (m, 3 H; H‐2, H‐3, H‐5), 3.65–3.57 (m, 1 H; H‐4); ^13^C NMR (100 MHz, D_2_O, 25 °C): *δ* = 181.2 (s, C = O), 140.4 (d, 2 × N*C*HCH), 117.4 (d, 2 × NCH*C*H), 92.4 (d, C‐1), 78.9 (d, C‐5), 75.7 (d, C‐3), 72.8 (d, C‐2), 68.9 (d, C‐4), 60.5 (t, C‐6); HRMS‐ES: calcd for C_11_H_16_NO_6_ [*M* + H]^+^: 258.0972, found: 258.0977.

##### Compound Data: 1‐(β‐D‐Glucopyranosyl)‐3‐Methylimidazolium Carbonate **2j**

General procedure A using D‐glucose **2a** (100 mg, 0.56 mmol), triethylamine (0.69 mL, 5.00 mmol), D_2_O (2 mL), 1‐methylimidazole **10j** (0.40 mL, 5.00 mmol), and CDMBI **1b** (362 mg, 1.67 mmol) gave compound **2j** (93 mg, 61%) as an orange oil. [α]_D_^20^ = +17 (*c* = 1.0 in water); IR (neat, cm^−1^): *ν*_max_ = 3149 (w) (C—H), 2912 (w) (C—H), 1620 (s) (C=C), 1020 (vs.) (C—O); ^1^H NMR (400 MHz, D_2_O, 25 °C): *δ* = 9.03 (s, <1 H [D_2_O exchange]; N = CHN), 7.70 (s, 1 H; C*H*CHNCH_3_), 7.52 (s, 1 H; CHC*H*NCH_3_), 5.49 (d, ^3^ *J*(H,H) = 8.4 Hz, 1 H; H‐1), 3.92 (s, 3 H; CH_3_), 3.90–3.89 (m, 1 H; H‐6’), 3.79 (dd, ^2^ *J*(H,H) = 12.5 Hz, ^3^ *J*(H,H) = 5.3 Hz, 1 H; H‐6), 3.73–3.65 (m, 3 H; H‐2, H‐3, H‐5), 3.62–3.55 (m, 1 H; H‐4); ^13^C NMR (100 MHz, D_2_O, 25 °C): *δ* = 163.5 (s, CO_3_), 135.7 (t, *J*_C‐D_ 31.6 Hz, N = CD‐N [D_2_O exchange]), 124.1 (d, CH*C*HNCH_3_), 120.2 (d, *C*HCHNCH_3_), 87.0 (d, C‐1), 79.1 (d, C‐3), 75.6 (d, C‐5), 73.0 (d, C‐2), 68.9 (d, C‐4), 60.5 (t, C‐6), 36.1 (q, CH_3_); HRMS‐ES: calcd for C_10_H_17_N_2_O_5_ [*M*]^+^: 245.1132, found: 245.1135.

##### Compound Data: 2‐(β‐D‐Glucopyranosyl) Isoquinolinium Carbonate **2l**

General procedure A using D‐glucose **2a** (100 mg, 0.56 mmol), triethylamine (0.69 mL, 5.00 mmol), D_2_O (2 mL), isoquinoline **10l** (0.59 mL, 5.00 mmol), and CDMBI **1b** (362 mg, 1.67 mmol) gave compound **2l** (31 mg, 17%) as a yellow solid. [α]_D_^20^ = +21 (*c* = 1.9 in water); IR (neat, cm^−1^): *ν*_max _ = 3195 (w) (C—H), 1641 (s) (C=C), 1019 (vs.) (C—O); ^1^H NMR (400 MHz, D_2_O, 25°C): *δ* = 9.94 (s, 1 H; NCH), 8.71 (dd, ^3^ *J*(H,H) = 7.0 Hz, ^4^ *J*(H,H) = 1.5 Hz; NC*H*CH), 8.53–8.45 (m, 2 H; 2 × Ar‐H), 8.32–8.22 (m, 2 H; 2 × Ar‐H), 8.10 (ddd, ^3^ *J*(H,H) = 8.3 Hz, 5.6 Hz, ^4^ *J*(H,H) = 2.5 Hz, 1 H; Ar‐H), 5.93 (d, ^3^ *J*(H,H) = 8.4 Hz, 1 H; H‐1), 4.04 (ad, ^2^ *J*(H,H) = 11.4 Hz, 1 H; H‐6’), 3.99–3.89 (m, 2 H; H‐5, H‐6), 3.85 (at, ^3^ *J*(H,H) = 9.0 Hz, 1 H; H‐3), 3.79 (at, ^3^ *J*(H,H) = 8.5 Hz, 1 H; H‐2), 3.77 (at, ^3^ *J*(H,H) = 9.1 Hz, 1 H; H‐4); ^13^C NMR (100 MHz, D_2_O, 25°C): *δ* = 161.6 (s, CO_3_), 139.1 (s, Ar‐C), 138.3, 131.8, 131.2, 130.9, 128.1, 127.5 (6 × d, 6 × Ar‐C), 127.2 (s, Ar‐C), 126.3 (d, Ar‐C), 95.1 (d, C‐1), 79.6 (d, C‐5), 75.5 (d, C‐3), 74.1 (d, C‐2), 68.8 (d, C‐4), 60.5 (t, C‐6); HRMS‐ES: calcd for C_15_H_18_NO_5_ [*M*]^+^: 292.1179, found: 292.1183.

##### Compound Data: N‐(β‐D‐Galactopyranosyl) Pyridinium Carbonate **3b**

General procedure A using D‐galactose **3a** (100 mg, 0.56 mmol), triethylamine (0.69 mL, 5.00 mmol), D_2_O (2 mL), pyridine (0.40 mL, 5.00 mmol), and CDMBI **1b** (362 mg, 1.67 mmol) gave compound **3b** (57 mg, 38%) as a yellow solid. [α]_D_^20^ = +18 (*c* = 0.1 in water); IR (neat, cm^−1^): ν_max_ = 3330 (br) (O—H), 2977 (w) (C—H), 1646 (m) (C=C), 1019 (vs.) (C—O); ^1^H NMR (400 MHz, D_2_O, 25 °C):^13^ *δ* = 9.08 (dd, ^3^ *J*(H,H) = 6.5 Hz, ^4^ *J*(H,H) = Hz, 2 H; 2 × NC*H*CH), 8.70 (tt, ^3^ *J*(H,H) = 7.6 Hz, ^4^ *J*(H,H) = 1.2 Hz, 1 H; NCHCHC*H*), 8.19 (t, ^3^ *J*(H,H) = 7.2 Hz, 2 H; 2 × NCHC*H*), 5.72 (d, ^3^ *J*(H,H) = 8.7 Hz, 1 H; H‐1), 4.14 (dd, ^3^ *J*(H,H) = 3.1 Hz, 0.8 Hz, 1 H; H‐4), 4.11 (ddd, ^3^ *J*(H,H) = 8.1 Hz, 4.3 Hz, 0.9 Hz, 1 H; H‐5), 3.98–3.79 (m, 4 H; H‐2, H‐3, H‐6, H‐6’); ^13^C NMR (100 MHz, D_2_O, 25 °C): *δ* = 148.3 (d, NCHCH*C*H), 142.2 (d, 2 × N*C*HCH), 128.2 (d, 2 × NCH*C*H), 95.8 (d, C‐1), 79.2 (d, C‐5), 72.5 (d, C‐3), 71.5 (d, C‐2), 68.5 (d, C‐4), 60.9 (t, C‐6); HRMS‐ES: calcd for C_11_H_16_NO_5_ [*M*]^+^: 242.1023, found: 242.1020.

##### Compound Data: N‐(α/β‐D‐Ribopyranosyl) Pyridinium Carbonate **4b**

General procedure A using D‐ribose **4a** (83 mg, 0.56 mmol), triethylamine (0.69 mL, 5.00 mmol), D_2_O (2 mL) pyridine (0.40 mL, 5.00 mmol), and CDMBI **1b** (362 mg, 1.67 mmol) gave compound **4b** (89 mg, 66%) as an orange oil (1:6 mixture of α and β anomers). IR (neat, cm^−1^): *ν*_max_ = 3217 (br) (O—H), 3172 (w) (C—H), 1628 (s) (C=C); β‐anomer: ^1^H NMR (400 MHz, D_2_O, 25 °C): *δ* = 9.03 (d, ^3^ *J*(H,H) = 6.7 Hz, 2 H; 2 × NC*H*CH), 8.70 (t, ^3^ *J*(H,H) = 7.8 Hz, 1 H; NCHCHC*H*), 8.18 (t, ^3^ *J*(H,H) = 7.1 Hz, 2 H; 2 × NCHC*H*), 5.84 (d, ^3^ *J*(H,H) = 9.0 Hz, 1 H; H‐1), 4.35 (bs, 1 H; H‐3), 4.19–4.09 (m, 2 H; H‐4, H‐5’), 3.95 (at, ^2^ *J*(H,H) = 12.3 Hz, 1 H; H‐5), 3.87 (dd, ^3^ *J*(H,H) = 9.0 Hz, 1.7 Hz, 1 H; H‐2); ^13^C NMR (100 MHz, D_2_O, 25 °C): *δ*  =  148.2 (d, NCHCH*C*H), 142.1 (d, 2 × N*C*HCH), 128.1 (d, 2 × NCH*C*H), 93.5 (d, C‐1), 71.4 (d, C‐2), 70.6 (d, C‐3), 65.7 (d, C‐4), 65.3 (t, C‐5); HRMS‐ES: calcd for C_10_H_14_NO_4_ [*M*]^+^: 212.0917, found: 212.0909.

##### Compound Data: N‐(α‐D‐Arabinopyranosyl) Pyridinium Carbonate **5b**

General procedure A using D‐arabinose **5a** (83 mg, 0.56 mmol), triethylamine (0.69 mL, 5.00 mmol), D_2_O (2 mL) pyridine (0.40 mL, 5.00 mmol), and CDMBI **1b** (362 mg, 1.67 mmol) gave compound **5b** (68 mg, 51%) as a yellow oil. [α]_D_^20^ = –56 (*c* = 1.0 in water); IR (neat, cm^−1^): ν_max_ = 3130 (br) (O—H), 2916 (w) (C—H), 1615 (s) (C=C), 1094 (vs.) (C—O); ^1^ H NMR (400 MHz, D_2_O, 25 °C): *δ* = 9.08 (d, ^3^ *J*(H,H) = 6.2 Hz, 2 H; 2 × NC*H*CH), 8.72 (t, ^3^ *J*(H,H) = 7.8 Hz, 1 H; NCHCHC*H*), 8.20 (t, ^3^ *J*(H,H) = 7.1 Hz, 2 H; 2 × NCHC*H*), 5.66 (d, ^3^ *J*(H,H) = 8.2 Hz, 1 H; H‐1), 4.31 (d, ^2^ *J*(H,H) = 13.0 Hz, 1 H; H‐5’), 4.18 (bs, 1 H; H‐4), 4.07 (d, ^2^ *J*(H,H) = 13.1 Hz, 1 H; H‐5), 3.98–3.81 (m, 2 H; H‐2, H‐3); ^13^C NMR (100 MHz, D_2_O, 25 °C): *δ* = 148.3 (d, NCHCH*C*H), 142.1 (d, 2 × N*C*HCH), 128.2 (d, 2 × NCH*C*H), 96.3 (d, C‐1), 72.2 (d, C‐3), 71.5 (d, C‐2), 70.2 (t, C‐5), 68.2 (d, C‐4); HRMS‐ES: calcd for C_10_H_14_NO_4_ [*M*]^+^: 212.0917, found: 212.0916.

##### Compound Data: N‐(4‐O‐α‐D‐Glucopyranosyl‐β‐D‐Glucopyranosyl) Pyridinium Carbonate **6b**

General procedure A using D‐maltose **6a** (190 mg, 0.56 mmol), triethylamine (0.69 mL, 5.00 mmol), D_2_O (2 mL), pyridine (0.40 mL, 5.00 mmol), and CDMBI **1b** (362 mg, 1.67 mmol) gave compound **6b** (69 mg, 29%) as a yellow solid. [α]_D_^20^ = +135 (*c* = 0.2 in water); IR (neat, cm^−1^): *ν*_max_ = 3265 (br) (O—H), 2923 (w) (C—H), 1631 (m) (C=C), 1020 (vs.) (C—O); ^1^H NMR (400 MHz, D_2_O, 25 °C): *δ* = 9.07 (d, ^3^ *J*(H,H) = 6.1 Hz, 2 H; 2 × NC*H*CH), 8.72 (t, ^3^ *J*(H,H) = 7.8 Hz, 1 H; NCHCHC*H*), 8.20 (t, ^3^ *J*(H,H) = 6.9 Hz, 2 H; 2 × NCHC*H*), 5.82 (d, ^3^ *J*(H,H) = 8.7 Hz, 1 H; H‐1a), 5.51 (d, ^3^ *J*(H,H) = 3.8 Hz, 1 H; H‐1b), 4.12–3.85 (m, 6 H; H‐3a, H‐4a, H‐5a, H‐6a, H‐6’a, H‐6’b), 3.84–3.67 (m, 4 H; H‐2a, H‐3b, H‐5b, H‐6b), 3.62 (dd, ^3^ *J*(H,H) = 9.9 Hz, 3.7 Hz, 1 H; H‐2b), 3.46 (at, ^3^ *J*(H,H) = 9.3 Hz, 1 H; H‐4b); ^13^C NMR (100 MHz, D_2_O, 25 °C): *δ* = 148.4 (d, NCHCH*C*H), 142.1 (d, 2 × N*C*HCH), 128.2 (d, 2 × NCH*C*H), 99.7 (d, C‐1b), 94.9 (d, C‐1a), 78.2 (d, C‐5a), 75.9 (d, C‐3a), 75.3 (d, C‐4a), 74.0, 72.9, 72.8 (3 × d, H‐2a, H‐3b, H‐5b), 71.7 (d, C‐2b), 69.4 (d, C‐4b), 60.6, 60.4 (2 × t, C‐6a, C‐6b); HRMS‐ES: calcd for C_17_H_26_NO_10_ [*M*]^+^: 404.1551, found: 404.1552.

##### Compound Data: N‐(4‐O‐β‐D‐Galactopyranosyl‐β‐D‐Glucopyranosyl) Pyridinium Carbonate **7b**

General procedure A using D‐lactose **7a** (190 mg, 0.56 mmol), triethylamine (0.69 mL, 5.00 mmol), D_2_O (2 mL), pyridine (0.40 mL, 5.00 mmol), and CDMBI **1b** (362 mg, 1.67 mmol) gave compound **7b** (51 mg, 21%) as a yellow solid. [α]_D_^20^ = +35 (*c* = 1.0 in water); IR (neat, cm^−1^): ν_max_ = 3209 (br) (O—H), 2877 (w) (C—H), 1616 (s) (C=C), 1067 (vs.) (C—O); ^1^ H NMR (400 MHz, D_2_O, 25 °C): *δ* = 9.08 (d, ^3^ *J*(H,H) = 5.5 Hz, 2 H; 2 × NC*H*CH), 8.73 (t, ^3^ *J*(H,H) = 7.8 Hz, 1 H; NCHCHC*H*), 8.21 (t, ^3^ *J*(H,H) = 7.1 Hz, 2 H; 2 × NCH*C*H), 5.85 (d, ^3^ *J*(H,H) = 8.8 Hz, 1 H; H‐1a), 4.55 (d, ^3^ *J*(H,H) = 7.8 Hz, 1 H; H‐1b), 4.12–3.91 (m, 6 H; H‐3a, H‐4a, H‐4b, H‐5b, H‐6b, H‐6’b), 3.87–3.68 (m, 5 H; H‐2a, H‐5a, H‐6a, H‐6’a, H‐3b), 3.61 (dd, ^3^ *J*(H,H) = 10.0 Hz, 7.7 Hz, 1 H; H‐2b); ^13^C NMR (100 MHz, D_2_O, 25 °C): *δ* = 148.4 (d, NCHCH*C*H), 142.2 (d, 2 × N*C*HCH), 128.2 (d, 2 × NCH*C*H), 103.0 (d, C‐1b), 94.9 (d, C‐1a), 78.4 (d, C‐5b), 77.0 (d, C‐4a), 75.5 (d, C‐5a), 74.1, 73.8 (2 × d, C‐2a, C‐3a), 72.6 (d, C‐3b), 71.0 (d, C‐2b), 68.6 (d, C‐4b), 61.2 (t, C‐6a), 59.7 (t, C‐6b); HRMS‐ES: calcd for C_17_H_26_NO_10_ [*M*]^+^: 404.1551, found: 404.1551.

##### Compound Data: N‐(2‐Acetamido‐2‐Deoxy‐β‐D‐Glucopyranosyl) Pyridinium Carbonate **8b**

General procedure B using *N*‐acetylglucosamine **8a** (50 mg, 0.23 mmol), triethylamine (0.28 mL, 2.04 mmol), water (1.3 mL), DMC **1a** (115 mg, 0.68 mmol), and then dry acetonitrile (5.0 mL), dry dimethylformamide (0.5 mL), powdered 4 Å molecular sieves (1.0 g), and pyridinium *p*‐toluenesulfonate (341 mg, 1.36 mmol) gave compound **8b** (50 mg, 70 %) as a yellow solid. [α]_D_^20^ = +9.7 (*c* = 1.0 in water); IR (neat, cm^−1^): *ν*_max_ = 3251 (br) (O—H), 2938 (w) (C—H), 1646 (s) (amide C=O), 1630 (s) (C=C), 1043 (vs.) (C—O); ^1^H NMR (400 MHz, D_2_O, 25 °C): *δ* = 9.09 (d, ^3^ *J*(H,H) = 6.1 Hz, 2 H; 2 × NC*H*CH), 8.70 (t, ^3^ *J*(H,H) = 7.9 Hz, 1 H; NCHCHC*H*), 8.17 (t, ^3^ *J*(H,H) = 7.0 Hz, 2 H; 2 × NCHC*H*), 5.83 (d, ^3^ *J*(H,H) = 9.3 Hz, 1 H; H‐1), 4.12 (at, ^3^ *J*(H,H) = 9.8 Hz, 1 H; H‐2), 4.03 (dd, ^2^ *J*(H,H) = 12.4 Hz, ^3^ *J*(H,H) = 1.6 Hz, 1 H; H‐6’), 3.97–3.81 (m, 3 H; H‐3, H‐5, H‐6), 3.78 (at, ^3^ *J*(H,H) = 9.3 Hz, 1 H; H‐4), 1.85 (s, 3 H, CH_3_); ^13^C NMR (100 MHz, D_2_O, 25 °C): *δ* = 174.7 (s, C=O), 148.7 (d, NCHCH*C*H), 142.3 (d, 2 × N*C*HCH), 128.1 (d, 2 × NCH*C*H), 94.2 (d, C‐1), 79.5 (d, C‐5), 72.6 (d, C‐3), 69.2 (d, C‐4), 60.4 (d, C‐6), 56.8 (d, C‐2), 21.3 (q, CH_3_); HRMS‐ES: calcd for C_13_H_19_N_2_O_5_ [*M*]^+^: 283.1289, found: 283.1281.

##### Compound Data: N‐(4‐O‐β‐D‐Mannopyranosyl‐2‐Acetamido‐2‐Deoxy‐β‐D‐Glucopyranosyl) Pyridinium Carbonate **9b**

General procedure B using 4‐*O*‐β‐D‐mannopyranosyl‐2‐acetamido‐2‐deoxy‐D‐glucose **9a** (25 mg, 0.065 mmol), triethylamine (0.082 mL, 0.59 mmol), water (0.36 mL), DMC **1a** (33 mg, 0.20 mmol), and then dry acetonitrile (1.5 mL), dry dimethylformamide (0.15 mL), powdered 4 Å molecular sieves (250 mg), and pyridinium *p*‐toluenesulfonate (98 mg, 0.39 mmol) gave compound **9b** (9 mg, 29%) as a white solid. [α]_D_^20^ = –4.7 (*c* = 0.3 in water); IR (neat, cm^−1^): *ν*_max_ = 3259 (br) (O—H), 2925 (w) (C—H), 1625 (s) (amide C=O), 1063 (s) (C—O); ^1^H NMR (400 MHz, D_2_O, 25 °C): *δ* = 9.10 (dd, ^3^ *J*(H,H) = 6.9 Hz, ^4^ *J*(H,H) = 1.3 Hz, 1 H; 2 × NC*H*CH), 8.71 (tt, ^3^ *J*(H,H) = 7.9 Hz, ^4^ *J*(H,H) = 1.4 Hz, 1 H; NCHCHC*H*), 8.17 (dd, ^3^ *J*(H,H) = 7.8 Hz, 6.7 Hz, 2 H; 2 × NCHC*H*), 5.86 (d, ^3^ *J*(H,H) = 9.2 Hz, 1 H; H‐1a), 4.88 (d, ^3^ *J*(H,H) = 1.0 Hz, 1 H; H‐1b), 4.19–4.14 (m, 1 H; H‐2a), 4.13 (dd, ^3^ *J*(H,H) = 3.3 Hz, 0.9 Hz, 1 H; H‐2b), 4.11–4.05 (m, 2 H; H‐3a, H‐4a), 4.03–3.90 (m, 4 H; H‐5a, H‐6a, H‐6’a, H‐6’b), 3.75 (dd, ^2^ *J*(H,H) = 12.3 Hz, ^3^ *J*(H,H) = 6.7 Hz, 1 H; H‐6b), 3.61 (at, ^3^ *J*(H,H) = 9.7 Hz, 1 H; H‐4b), 3.70 (dd, ^3^ *J*(H,H) = 9.6 Hz, 3.1 Hz, 1 H; H‐3b), 3.48 (ddd, ^3^ *J*(H,H) = 9.4 Hz, 6.8 Hz, 2.3 Hz, 1 H; H‐5b), 1.85 (s, 3 H; CH_3_); ^13^C NMR (100 MHz, D_2_O, 25 °C): *δ* = 174.7 (s, C=O), 148.7 (d, NCHCH*C*H), 142.3 (d, 2 × N*C*HCH), 128.1 (d, 2 × NCH*C*H), 100.2 (d, C‐1b), 94.0 (d, C‐1a), 78.1 (d, C‐5a), 77.9 (d, C‐4a), 76.5 (d, C‐5b), 72.8 (d, C‐3b), 71.2 (d, C‐3a), 70.6 (d, C‐2b), 66.7 (d, C‐4b), 61.0 (t, C‐6b), 59.9 (t, C‐6a), 56.3 (d, C‐2a), 21.3 (q, CH_3_); HRMS‐ES: calcd for C_19_H_29_N_2_O_10_ [*M*]^+^: 445.1817.

## Conflict of Interest

The authors declare no conflict of interest.

## Supporting information

Supplementary Material

## Data Availability

The data that support the findings of this study are available in the supplementary material of this article.
