# Supplementary material for: One‐Step Aqueous Synthesis of Glycosyl Pyridinium Salts, Electrochemical Study, and Assessment of Utility as Precursors of Glycosyl Radicals Using Photoredox Catalysis
Source: ChemistryOpen. 2025 Apr 21;14(10):e202500183. doi: 10.1002/open.202500183 (PMC12518051; doi:10.1002/open.202500183)
Supplement: Supplementary file 1 — Supplementary Material [file OPEN-14-e202500183-s001.pdf]

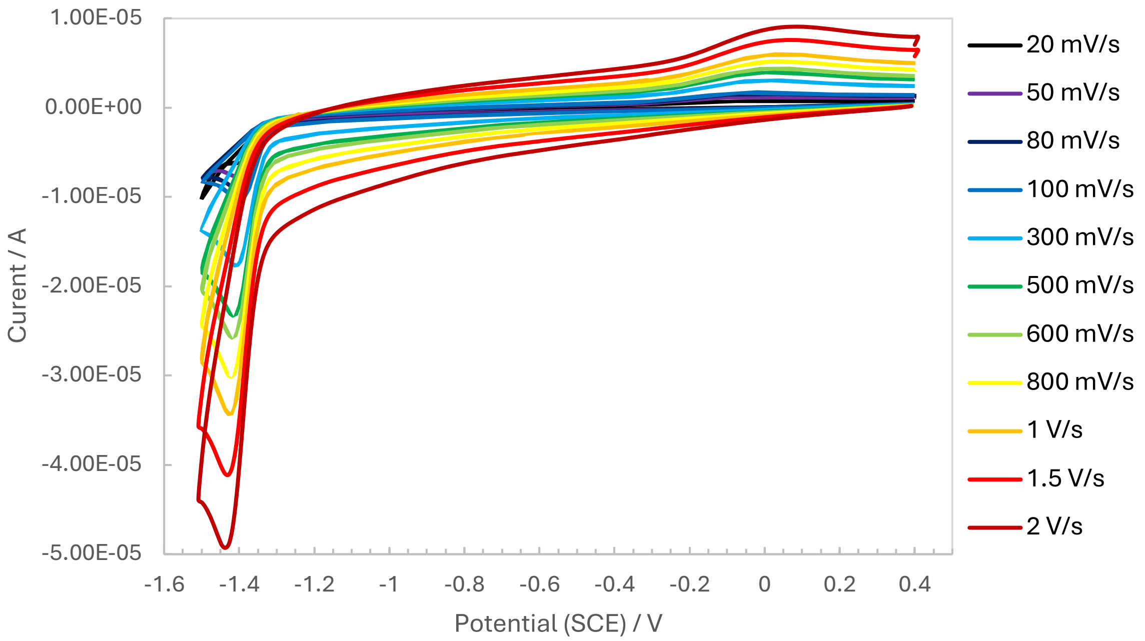

Figure S1: Cyclic voltammogram of 0.87 mM **2d** in aqueous 0.1 M Na_2SO_4 (10 mL, N_2 sparged); scan rates between 20 mV s^{-1} and 2 V s^{-1}

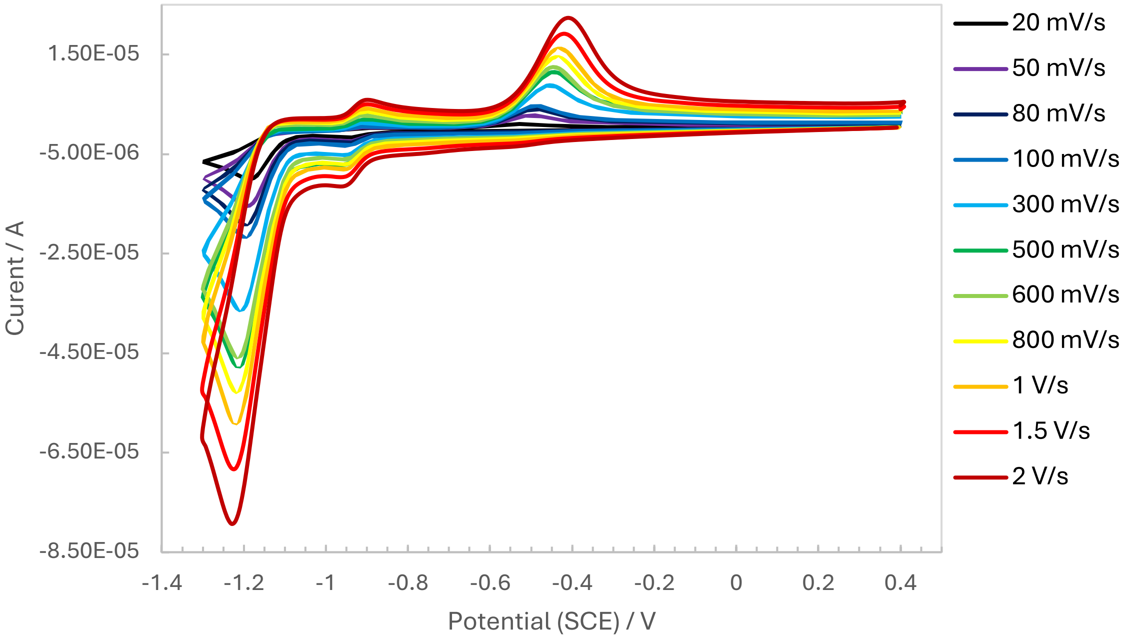

Figure S2: Cyclic voltammogram of 6.3 mM **2g** in aqueous 0.1 M Na_2SO_4 (10 mL, N_2 sparged); scan rates between 20 mV s^{-1} and 2 V s^{-1}

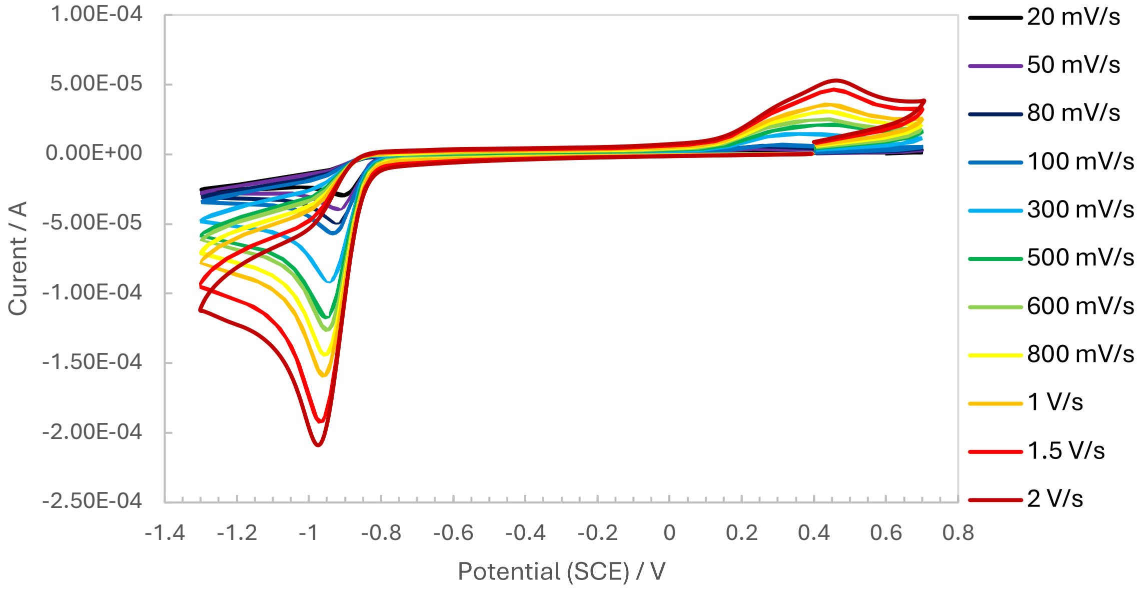

Figure S3: Cyclic voltammogram of 6.5 mM **2h** in aqueous 0.1 M Na_2SO_4 (10 mL, N_2 sparged); scan rates between 20 mV s^{-1} and 2 V s^{-1}

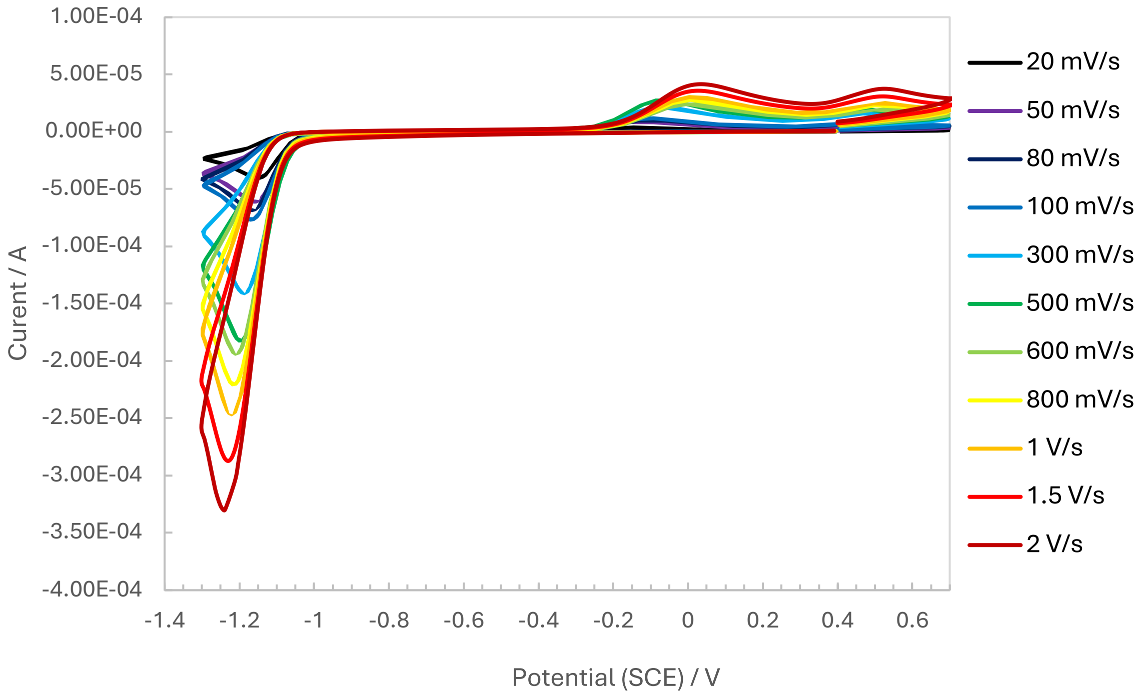

Figure S4: Cyclic voltammogram of 9.3 mM **2l** in aqueous 0.1 M Na_2SO_4 (10 mL, N_2 sparged); scan rates between 20 mV s^{-1} and 2 V s^{-1}

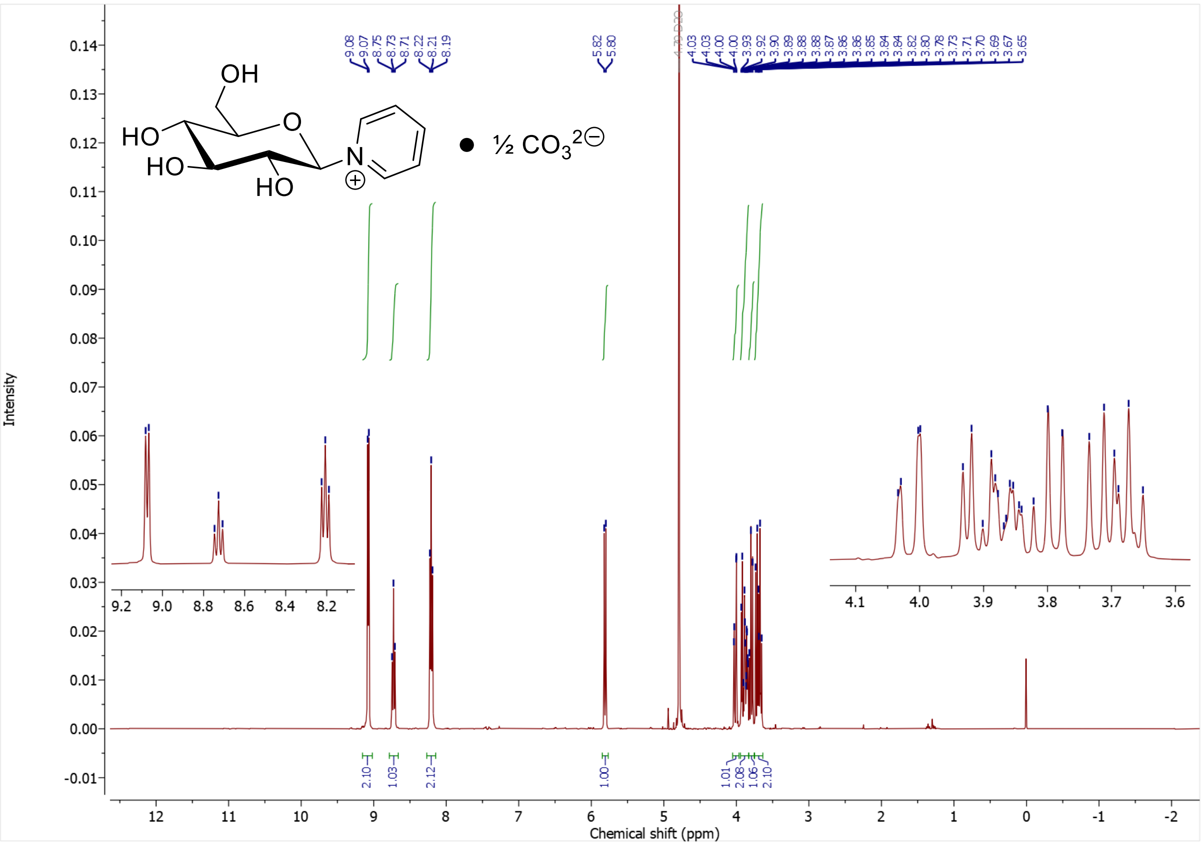

Figure S5: ^{1}H NMR spectrum of **2b**

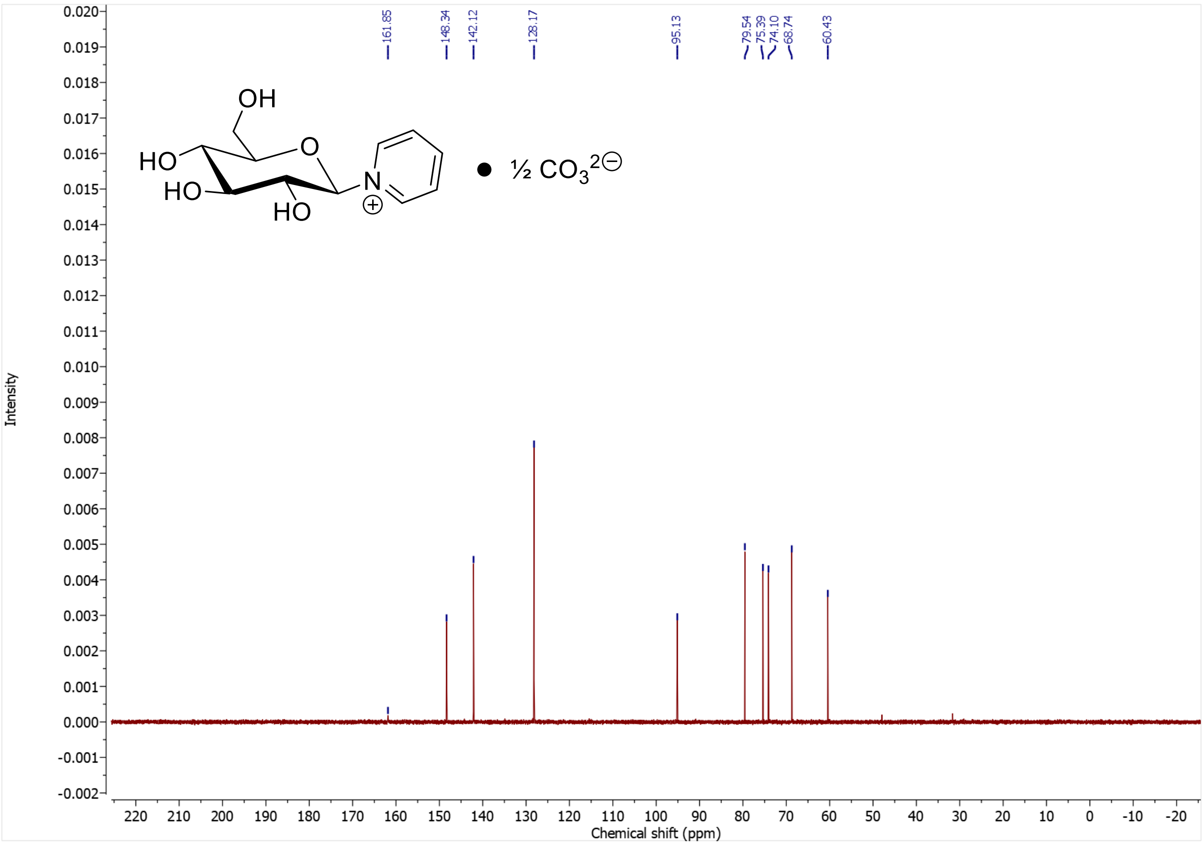

Figure S6: ^{13}C NMR spectrum of **2b**

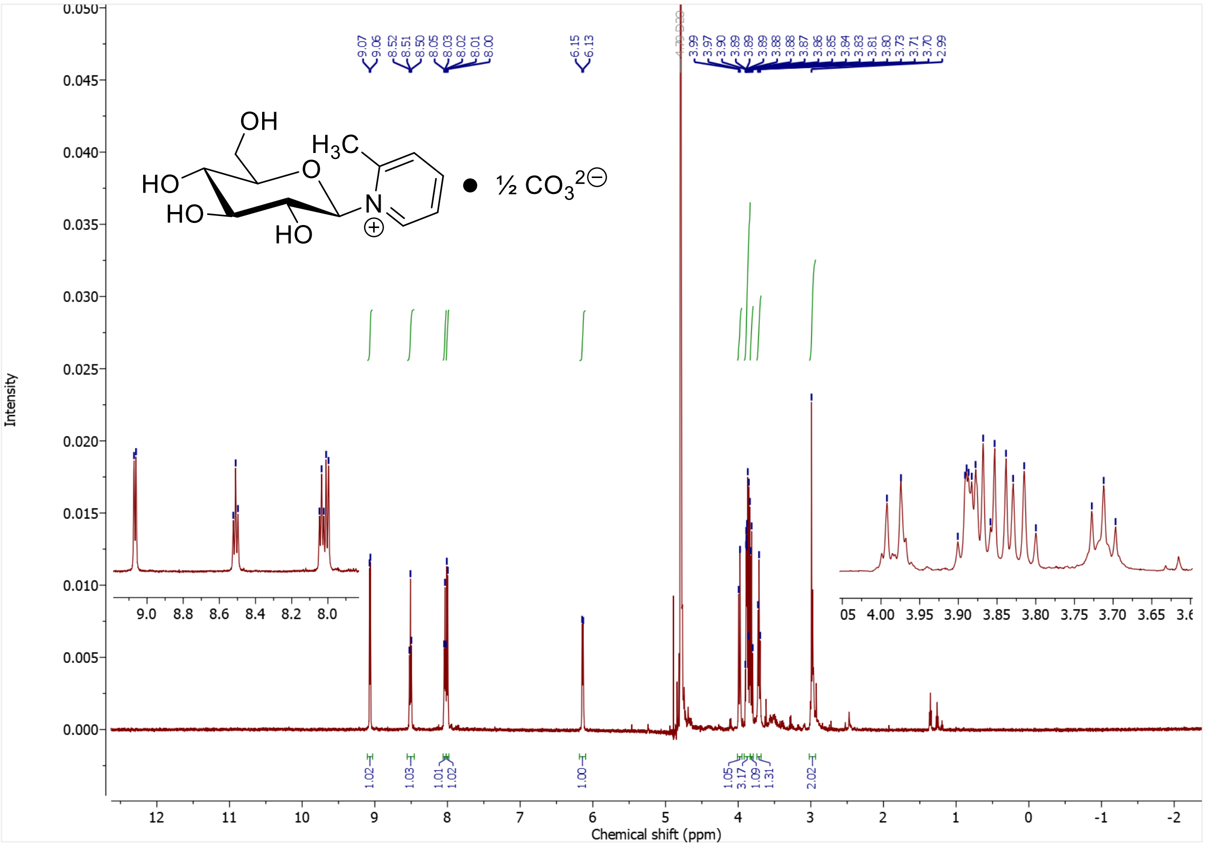

Figure S7: ^{1}H NMR spectrum of **2d**

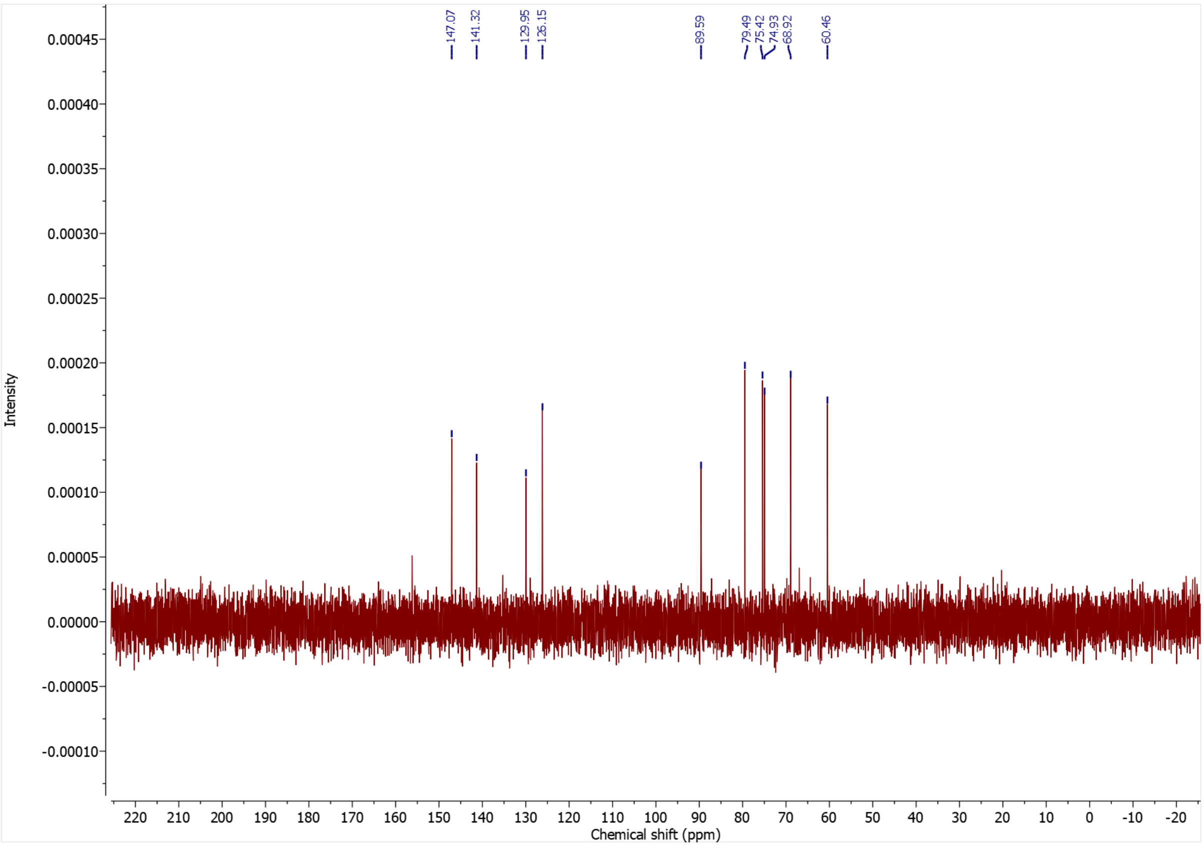

Figure S8: ^{13}C NMR spectrum of **2d**

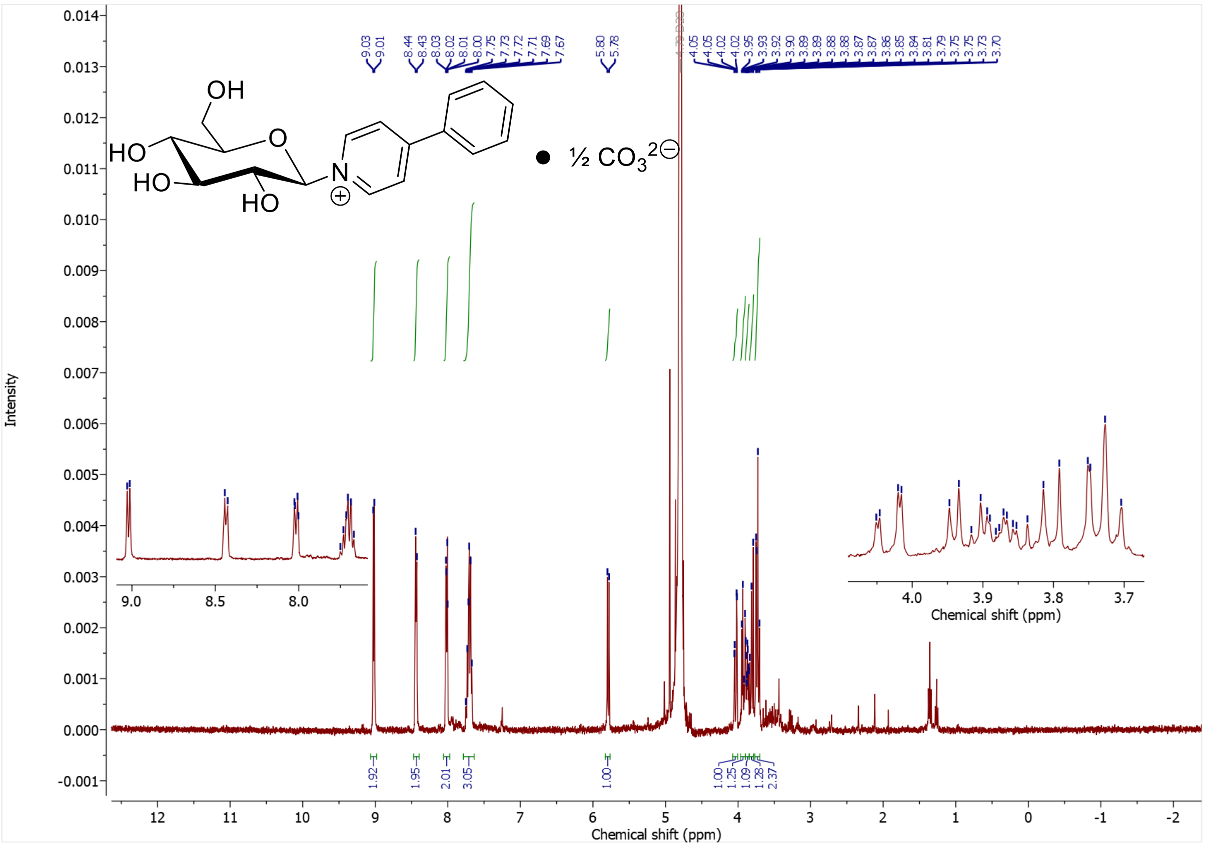

Figure S9: ^{1}H NMR spectrum of **2g**

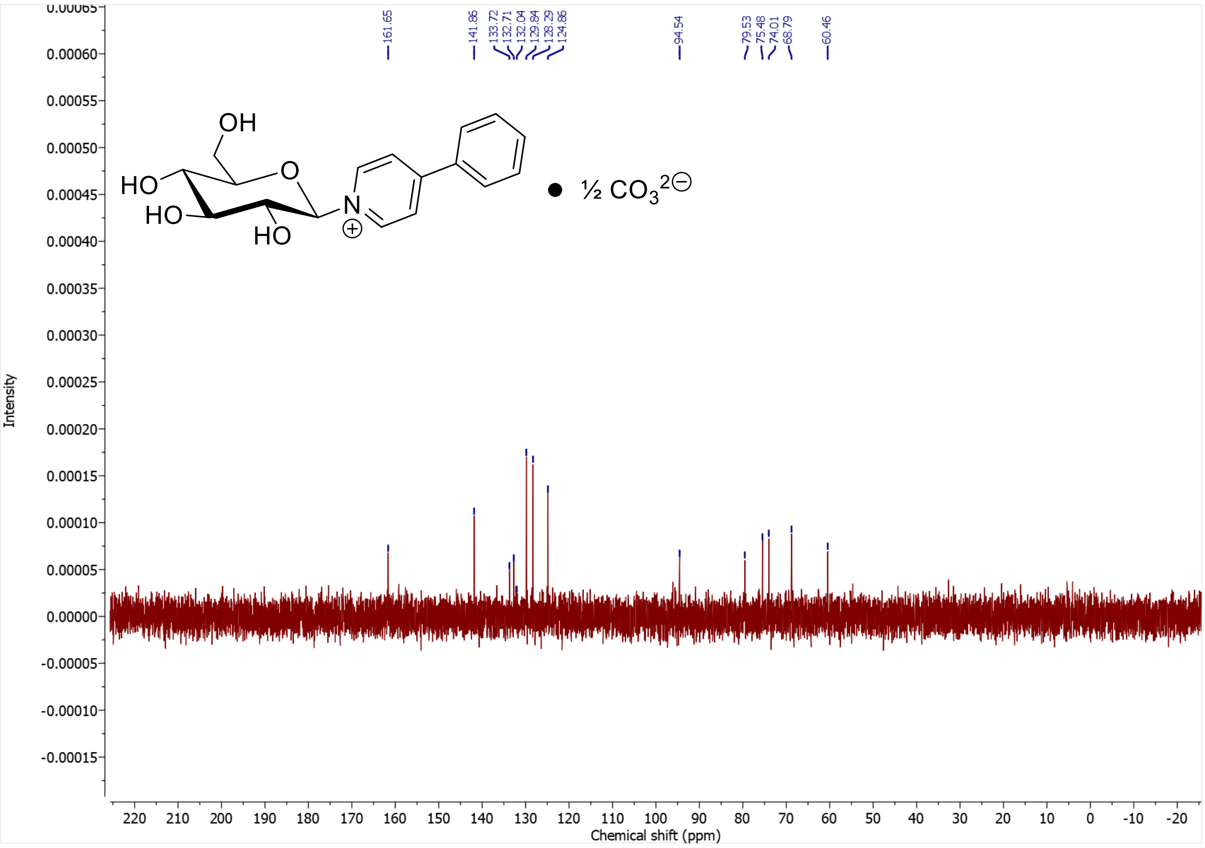

Figure S10: ^{13}C NMR spectrum of **2g**

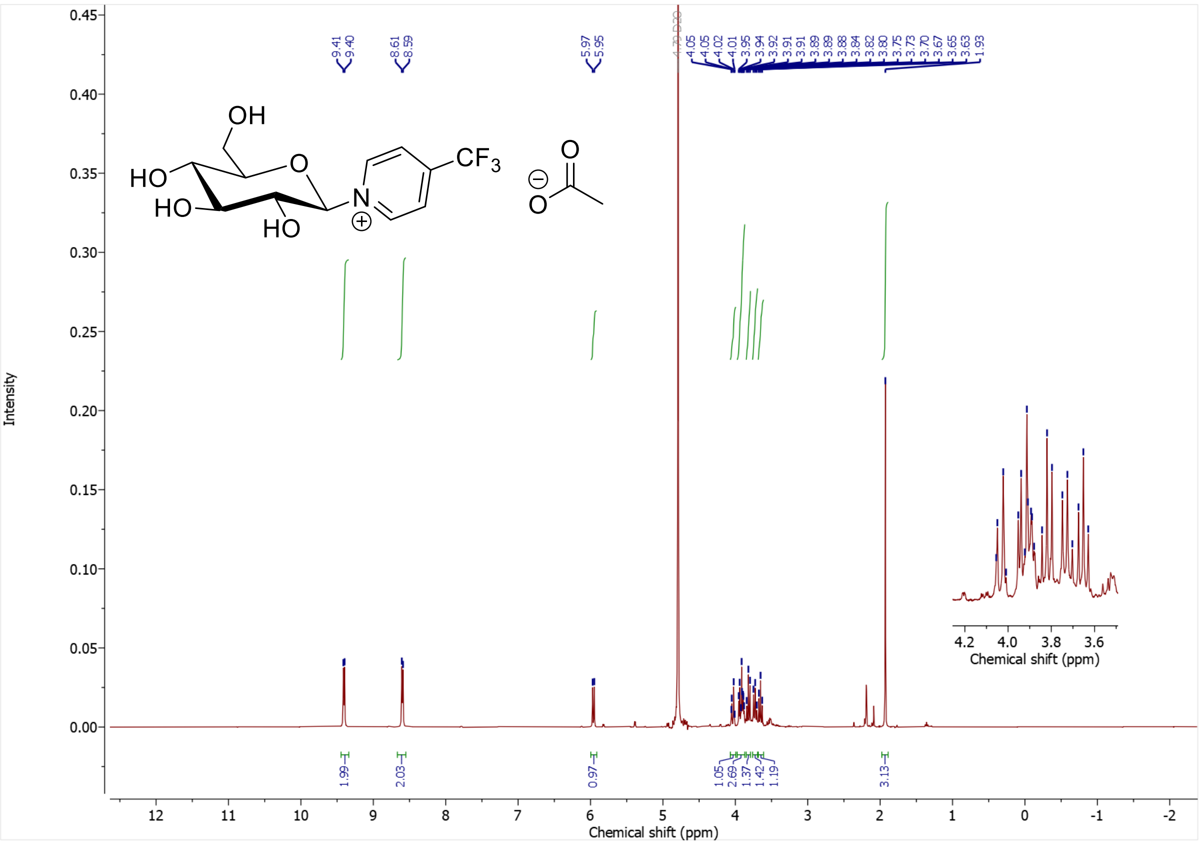

Figure S11: ^{1}H NMR spectrum of **2h**

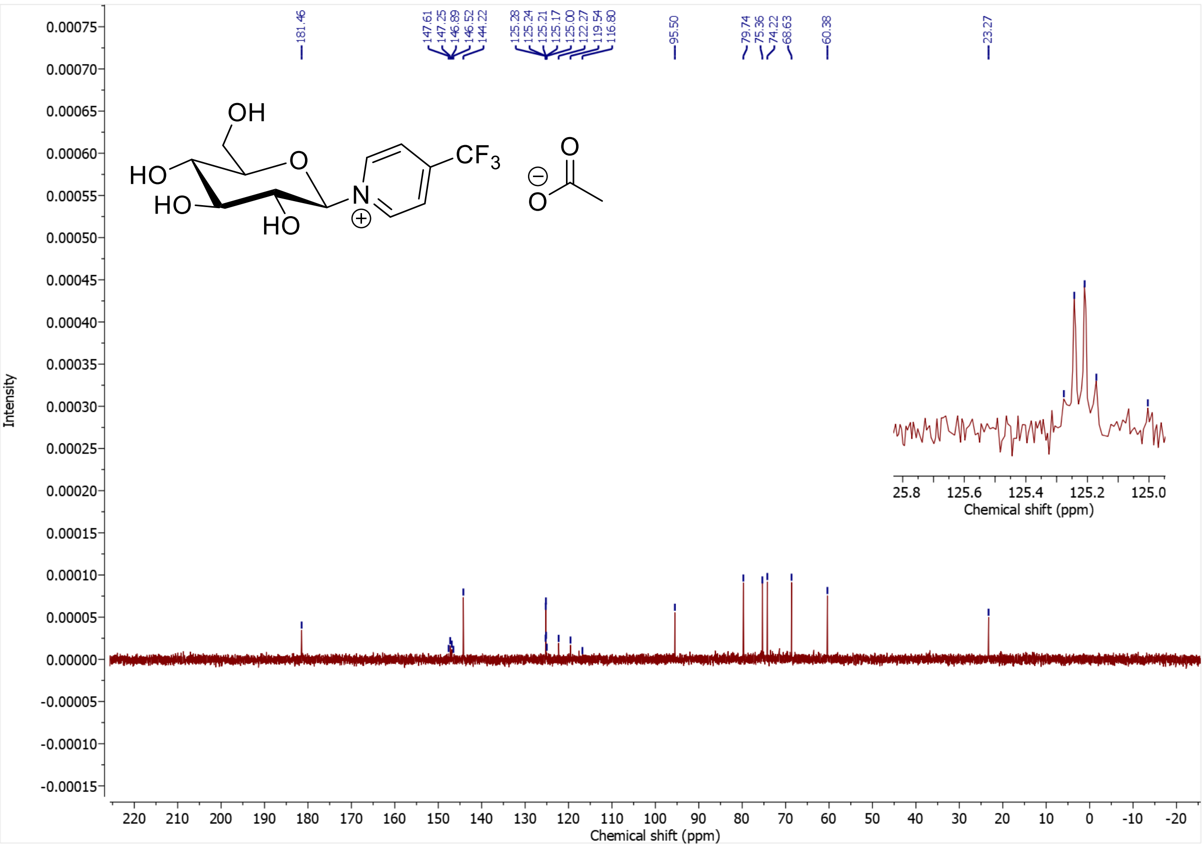

Figure S12: ^{13}C NMR spectrum of **2h**

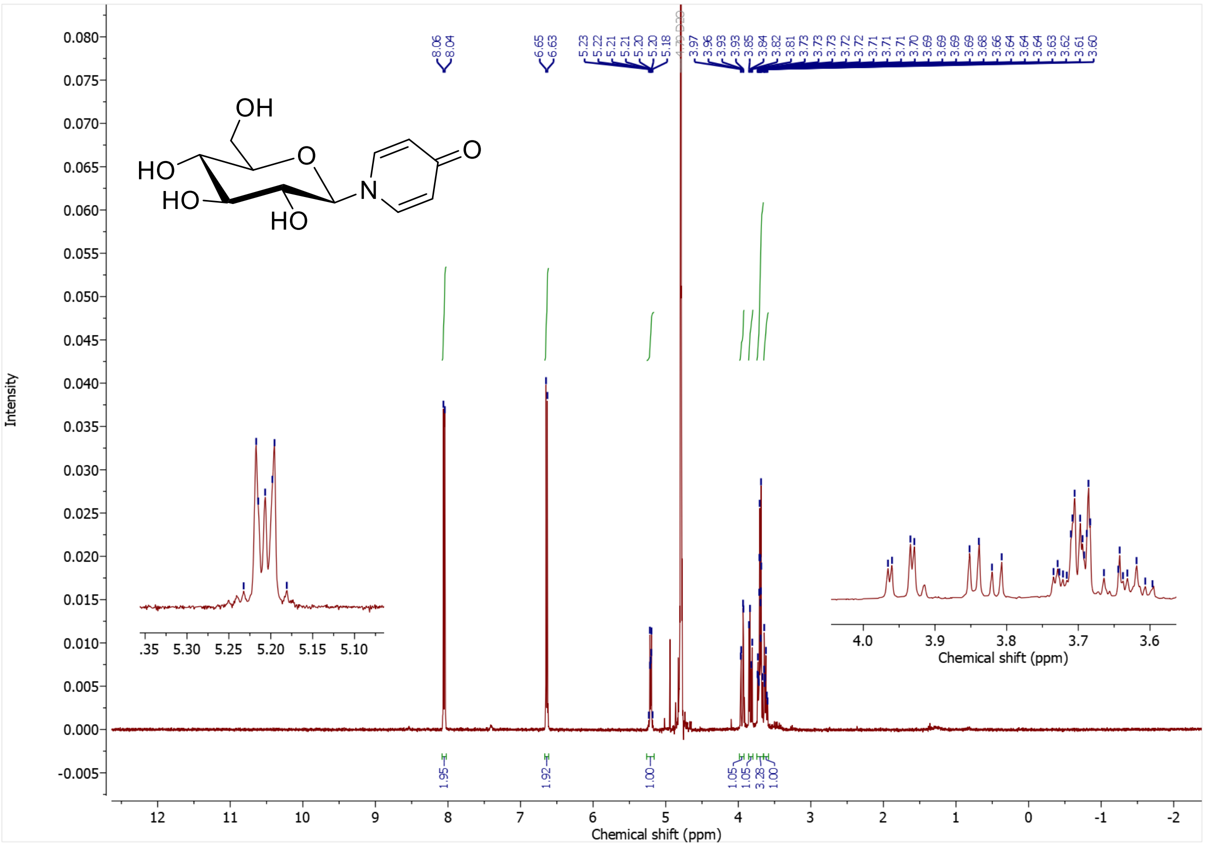

Figure S13: ^{1}H NMR spectrum of **2i**

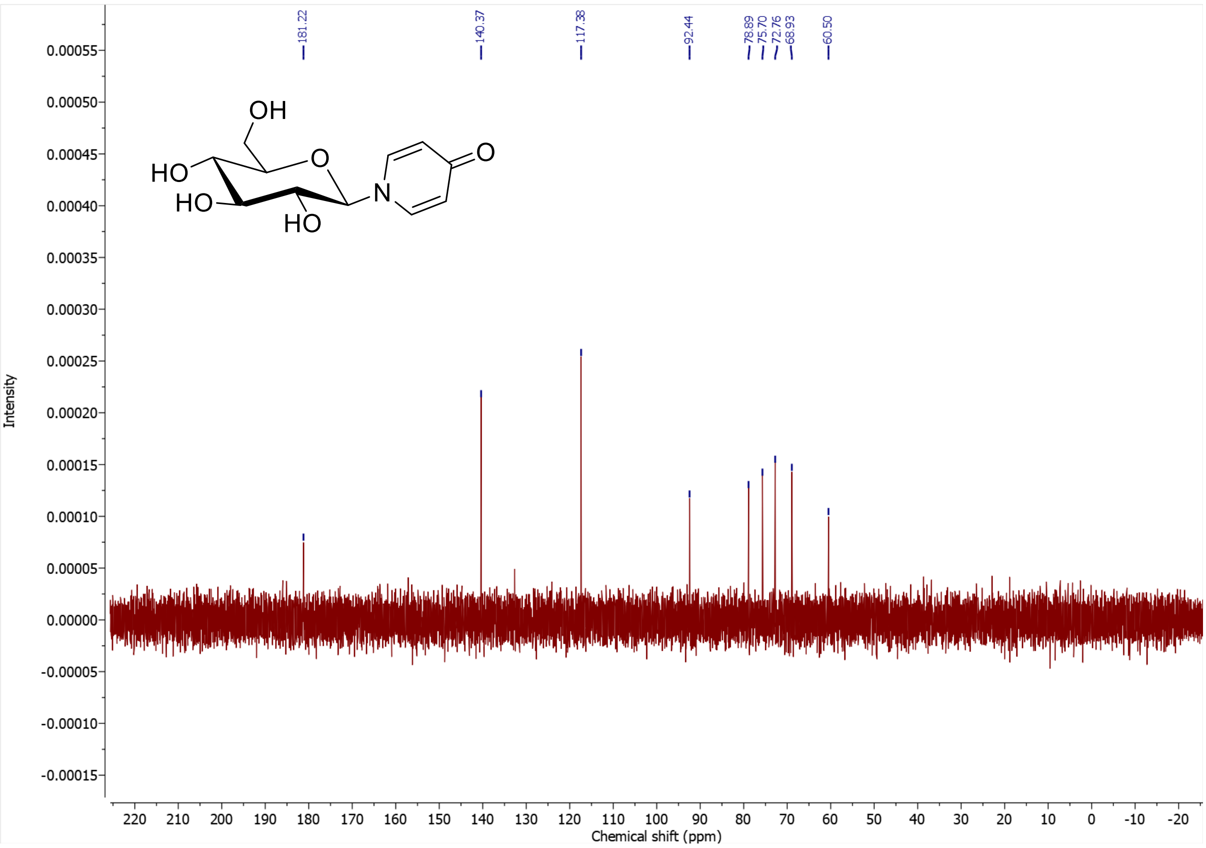

Figure S14: ^{13}C NMR spectrum of **2i**

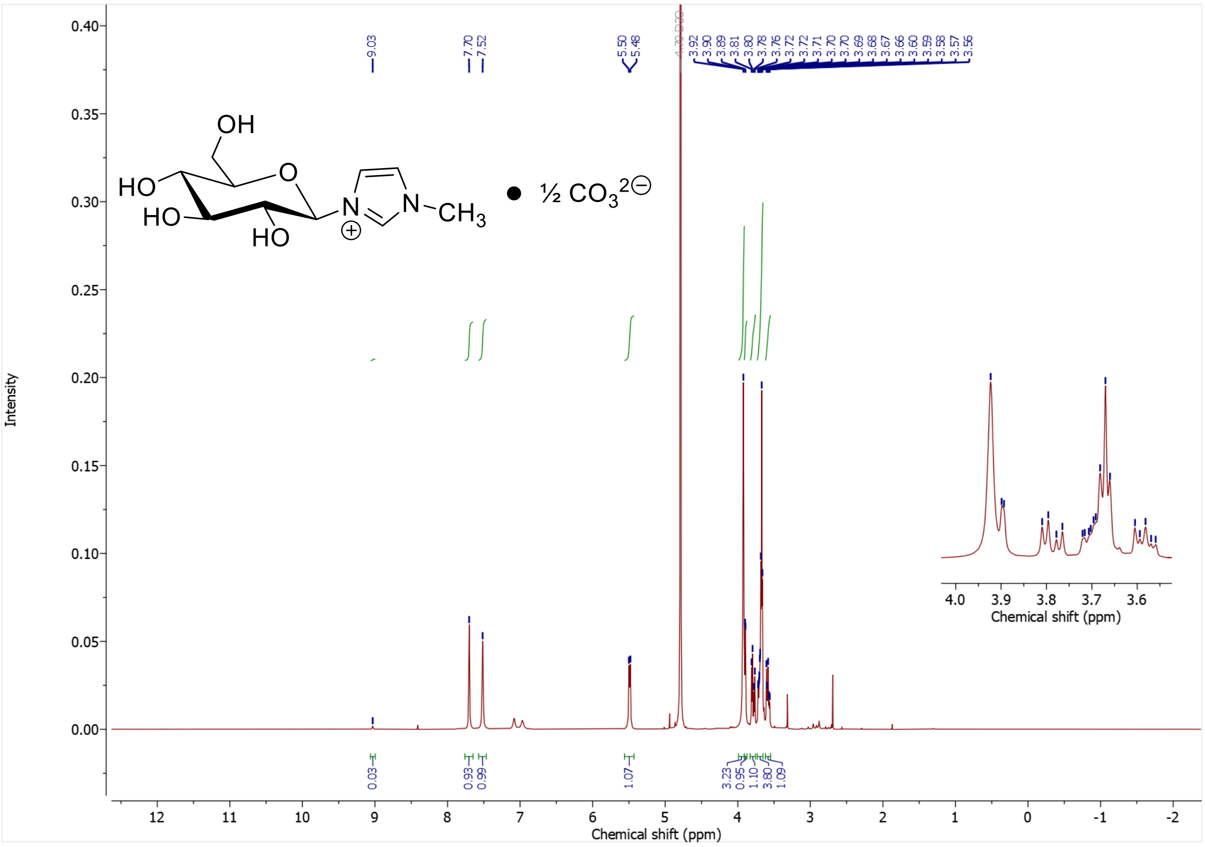

Figure S15: ^{1}H NMR spectrum of **2j**

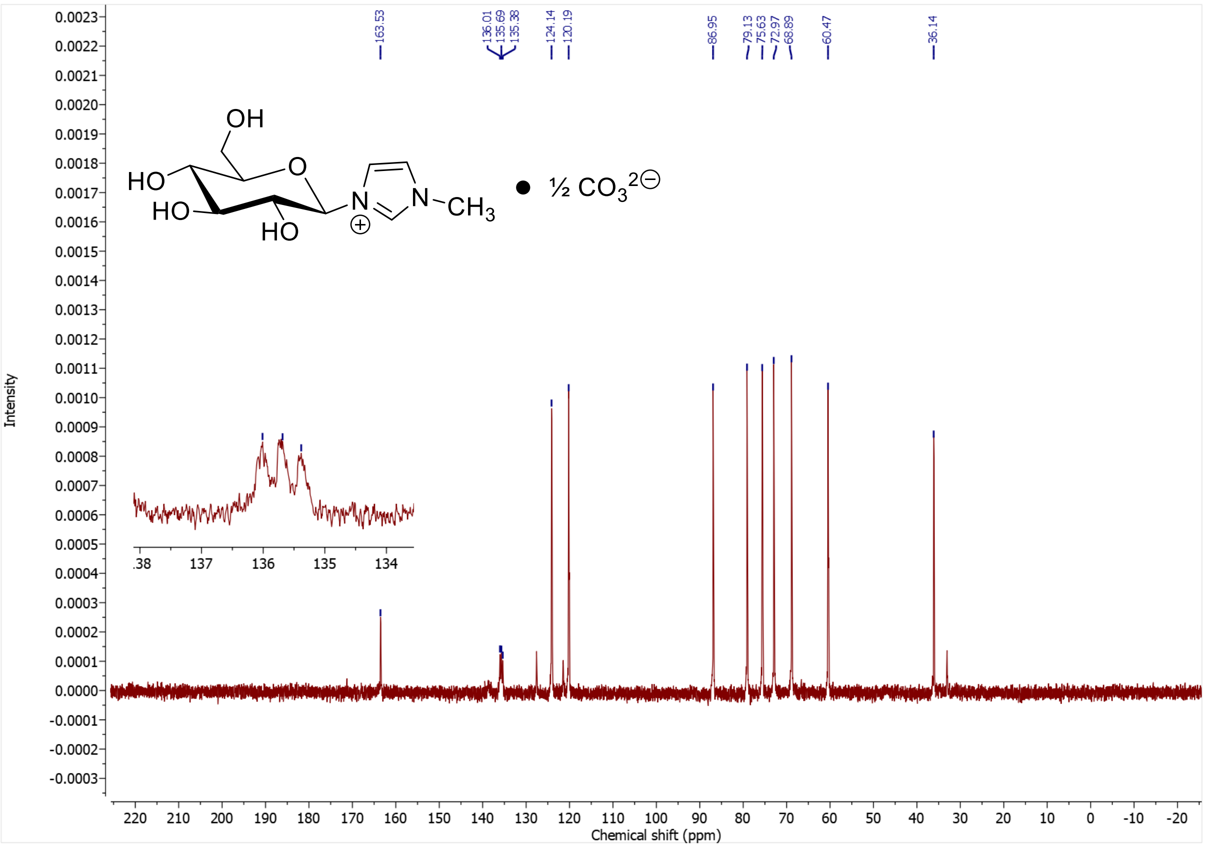

Figure S16: ^{13}C NMR spectrum of **2j**

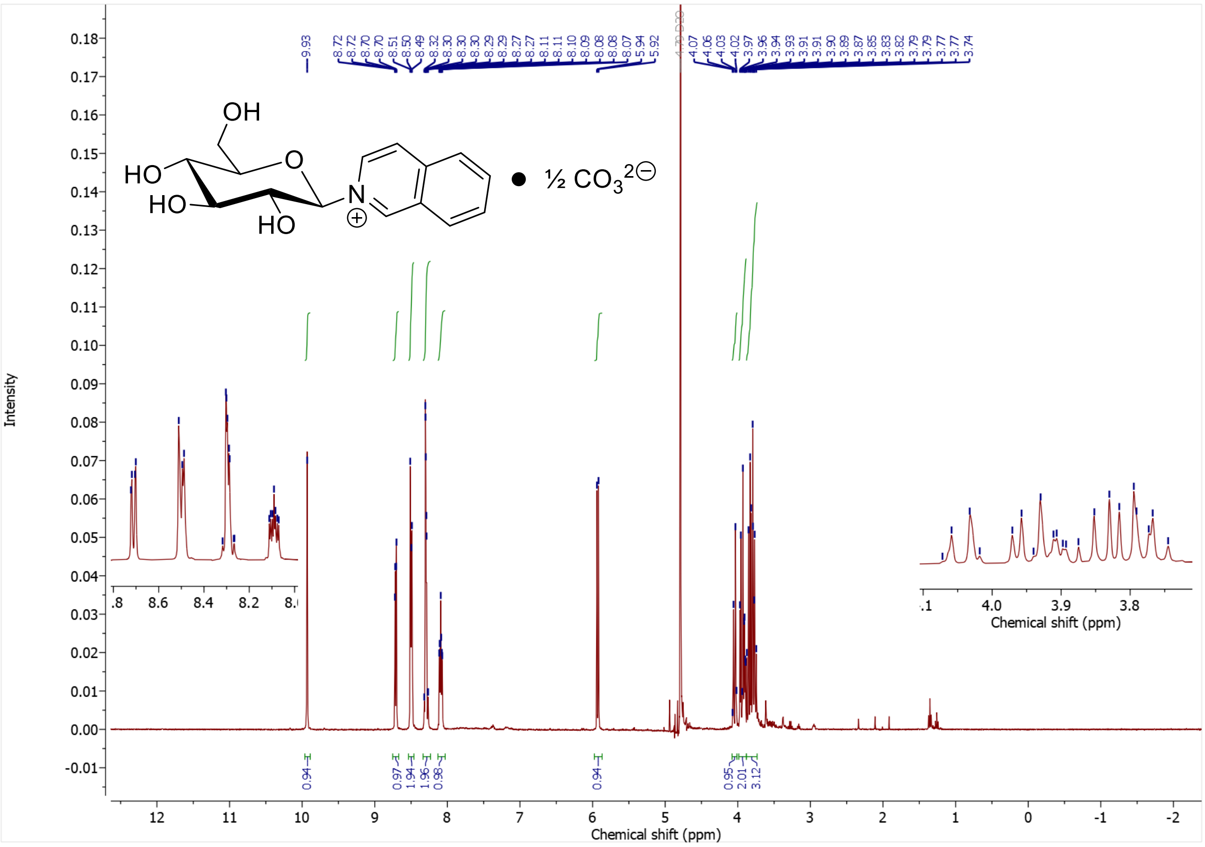

Figure S17: ^{1}H NMR spectrum of **2l**

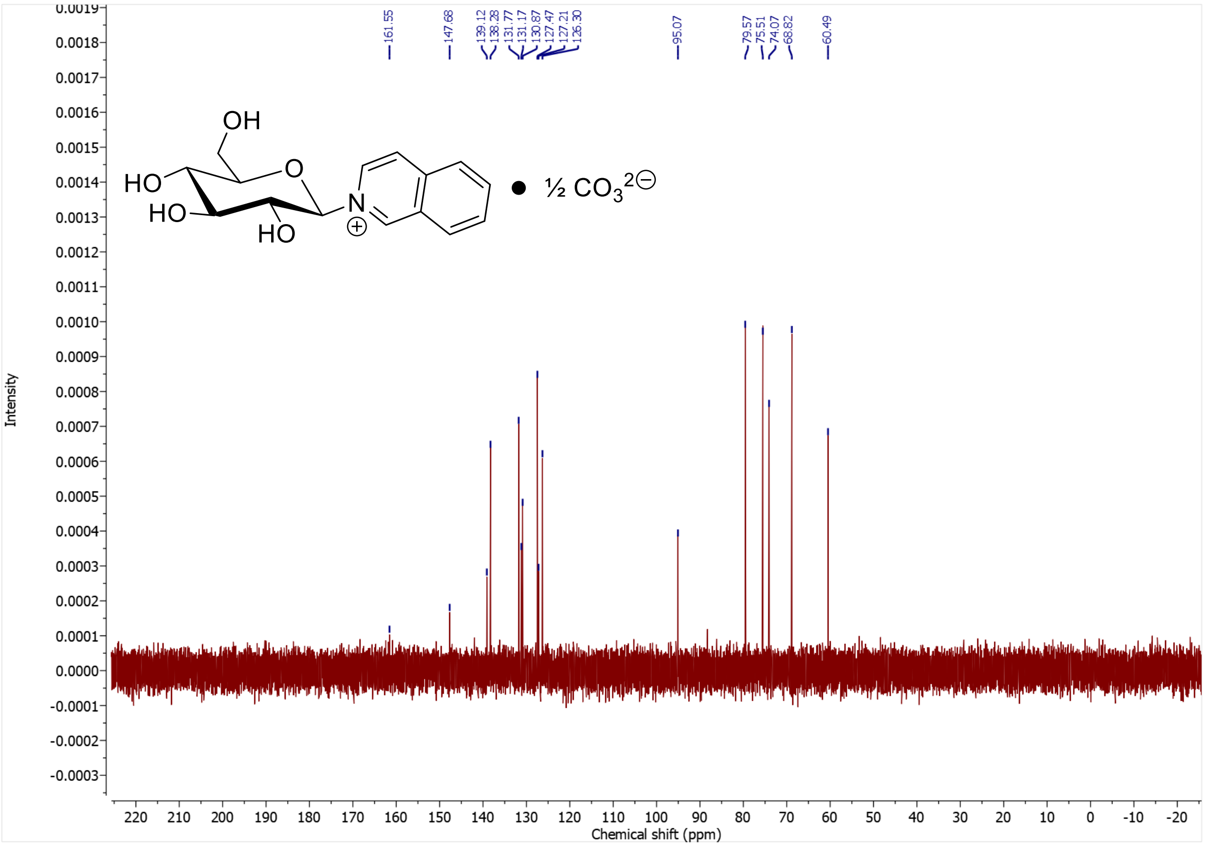

Figure S18: ^{13}C NMR spectrum of **2l**

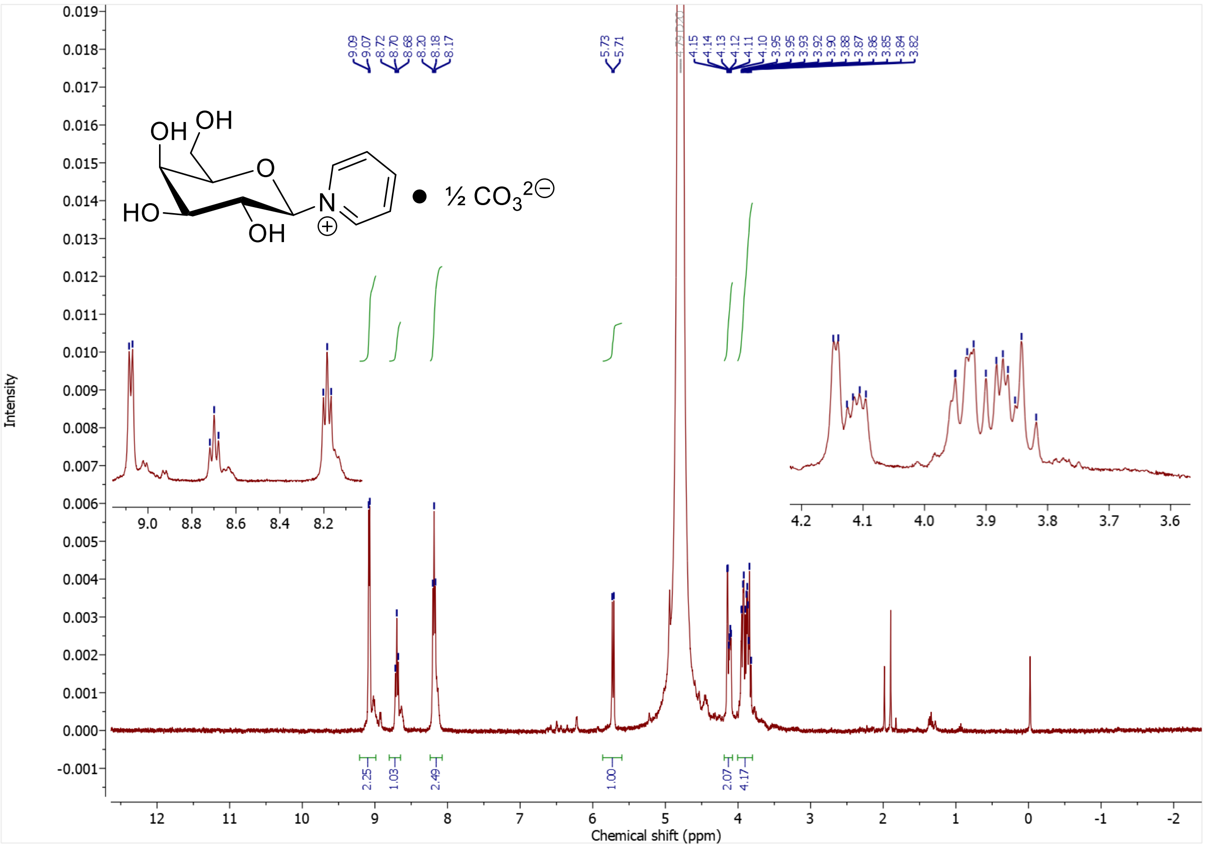

Figure S19: ^{1}H NMR spectrum of **3b**

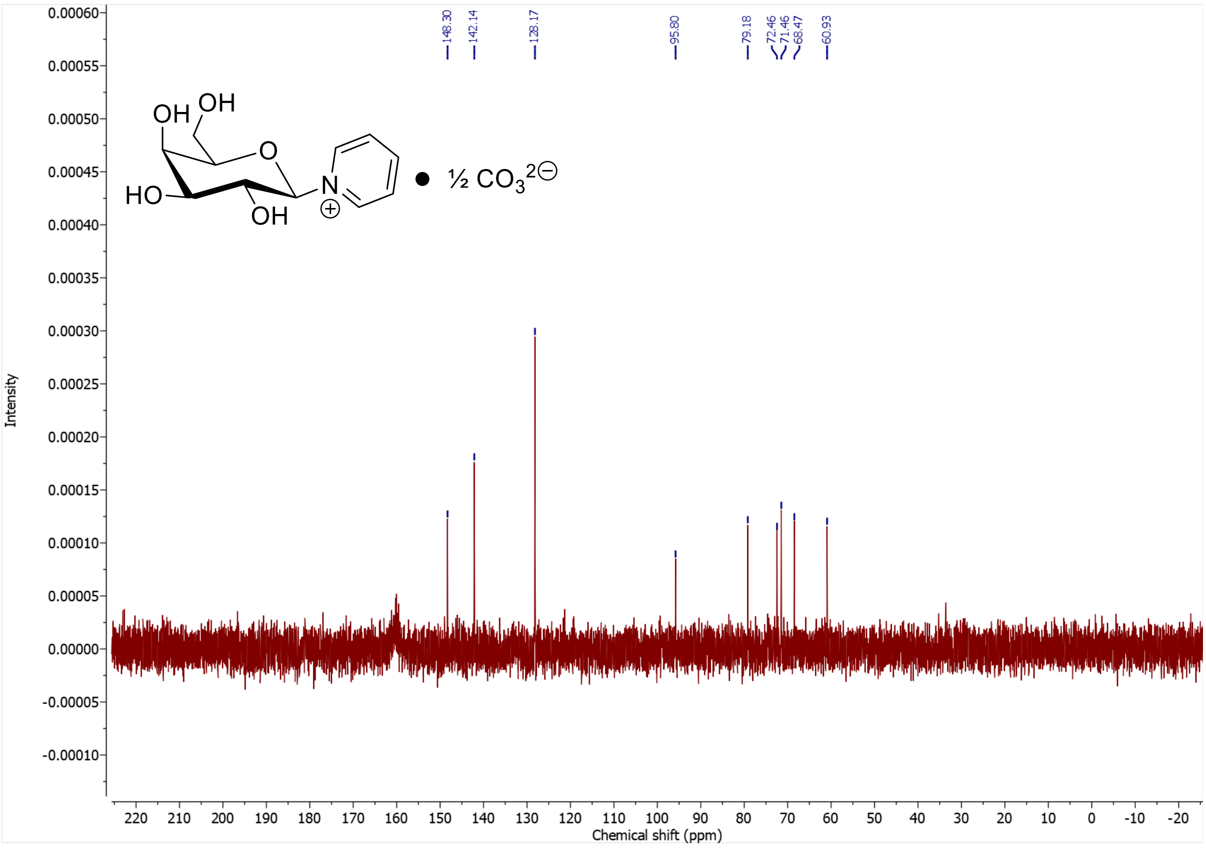

Figure S20: ^{13}C NMR spectrum of **3b**

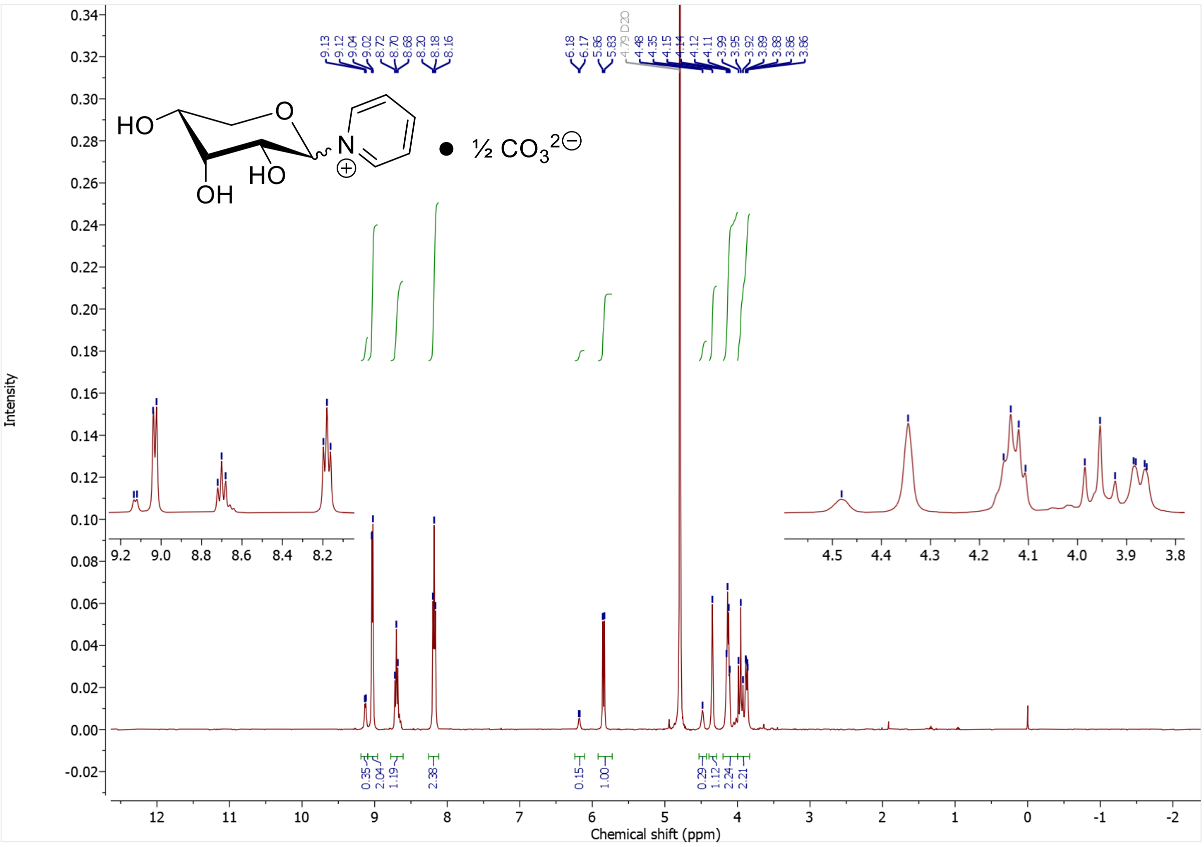

Figure S21: ^{1}H NMR spectrum of **4b**

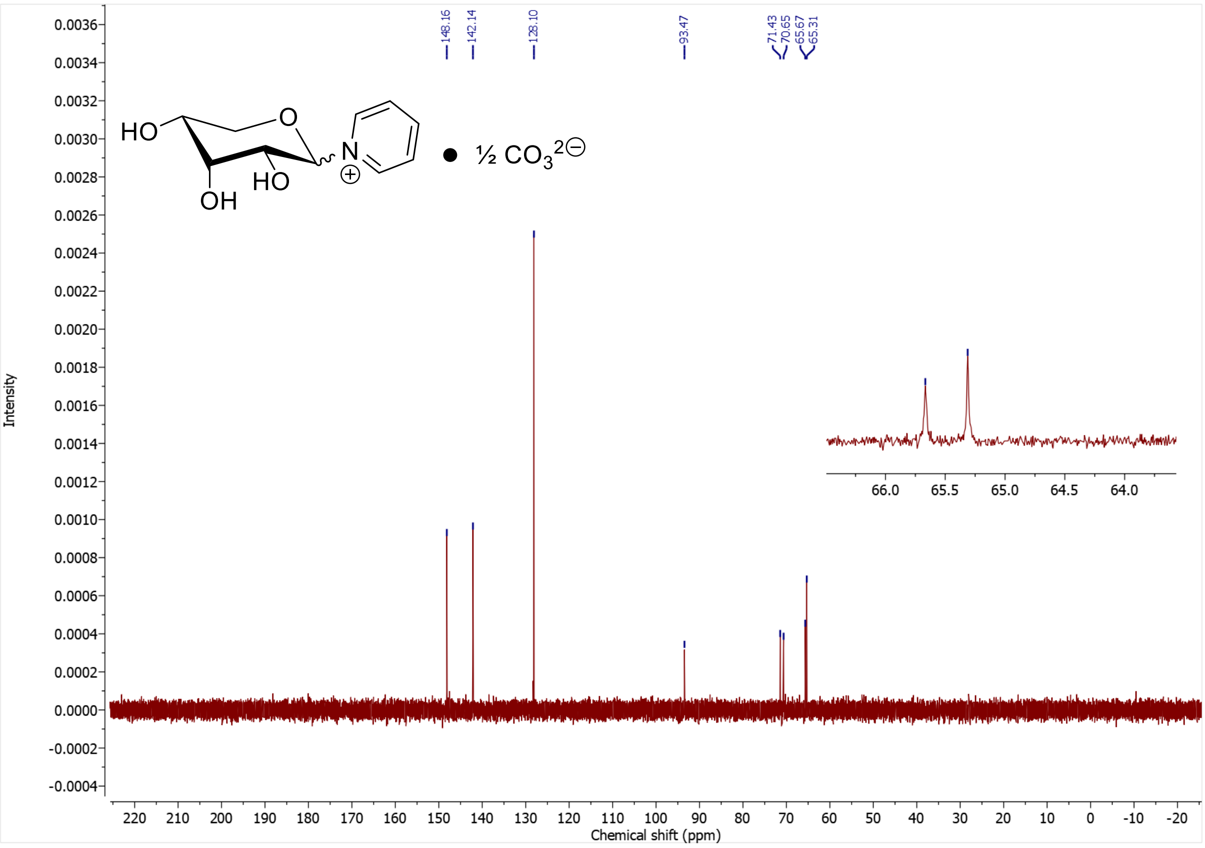

Figure S22: ^{13}C NMR spectrum of **4b**

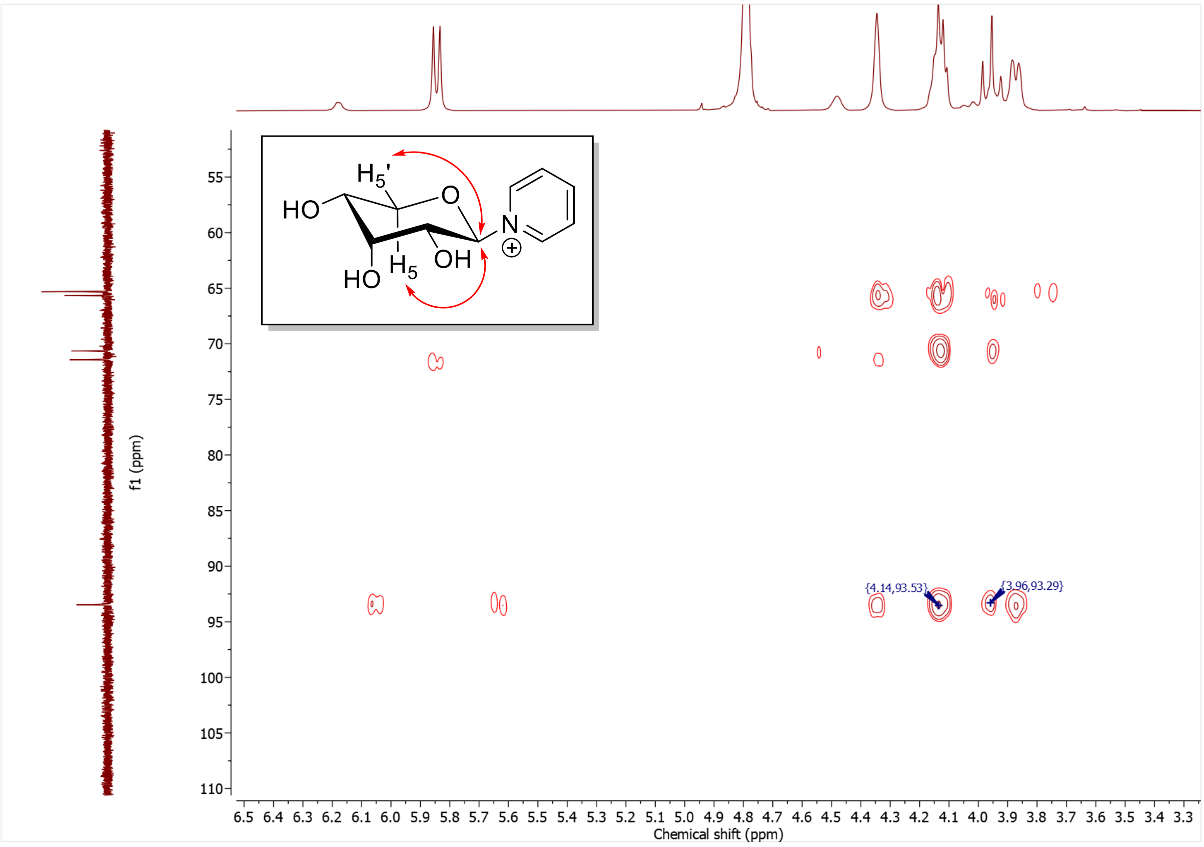

Figure S23: ^{1}H-^{13}C HMBC spectrum of **4b**

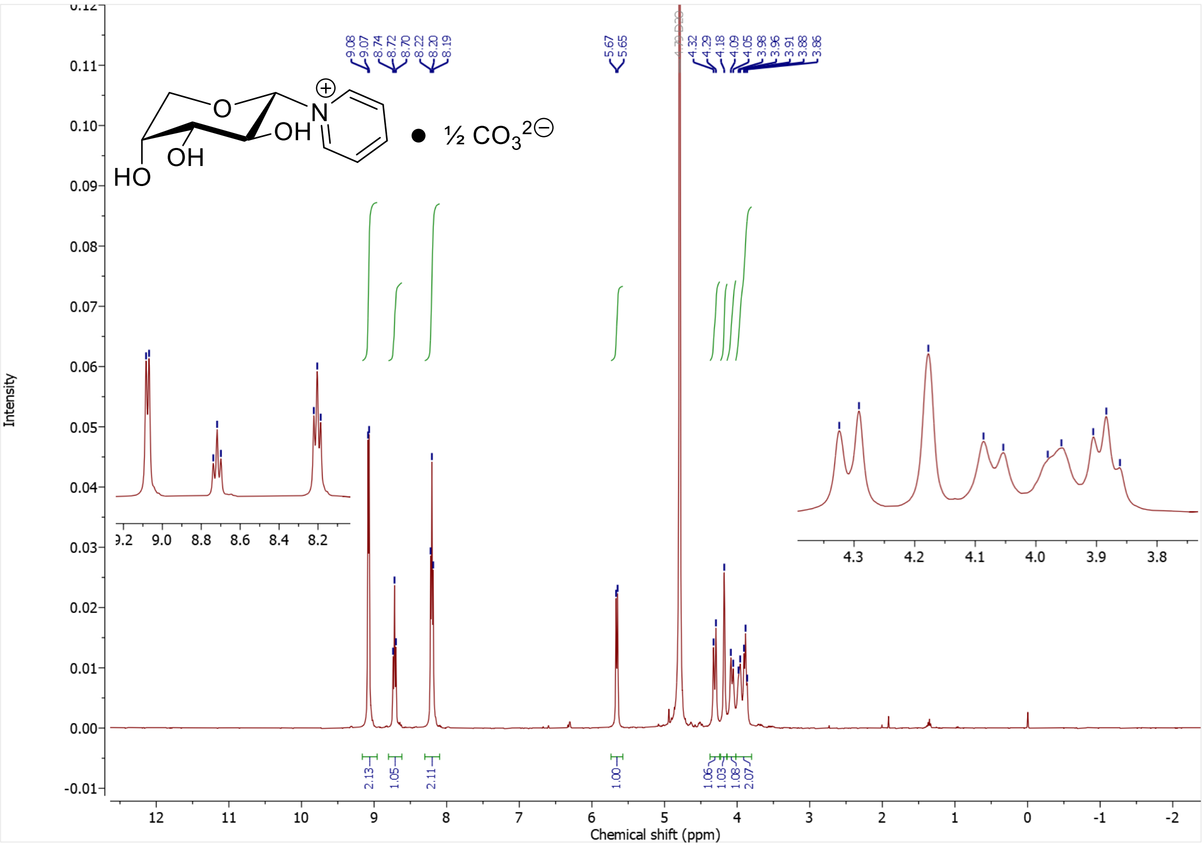

Figure S24: ^{1}H NMR spectrum of **5b**

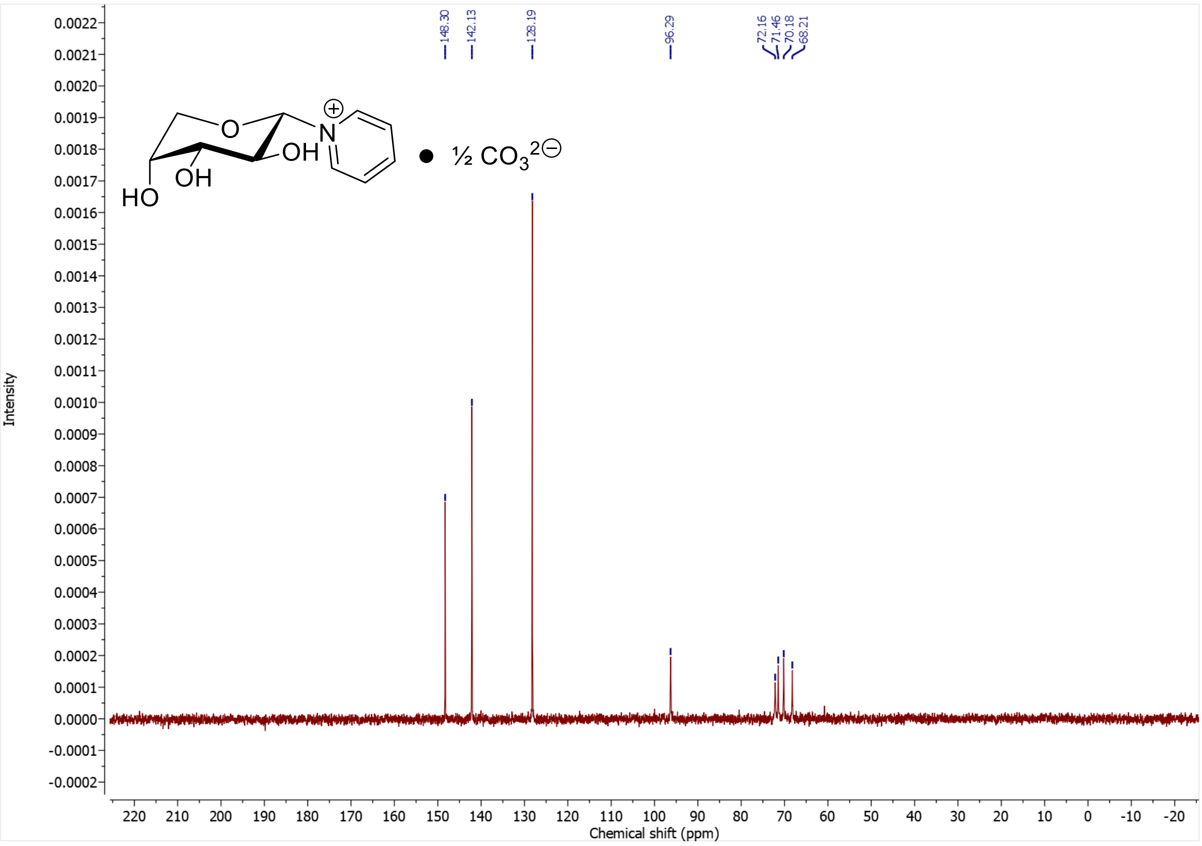

Figure S25: ^{13}C NMR spectrum of **5b**

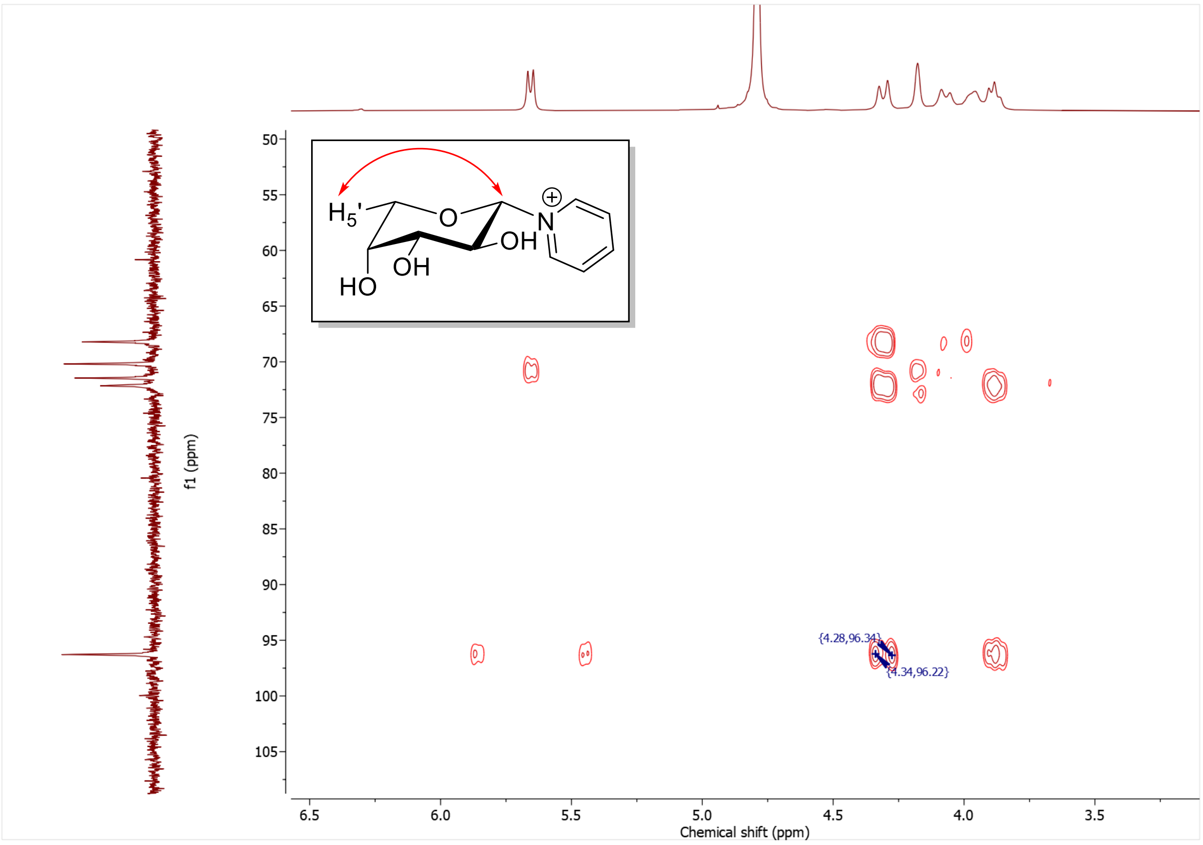

Figure S26: ^{1}H-^{13}C HMBC spectrum of **5b**

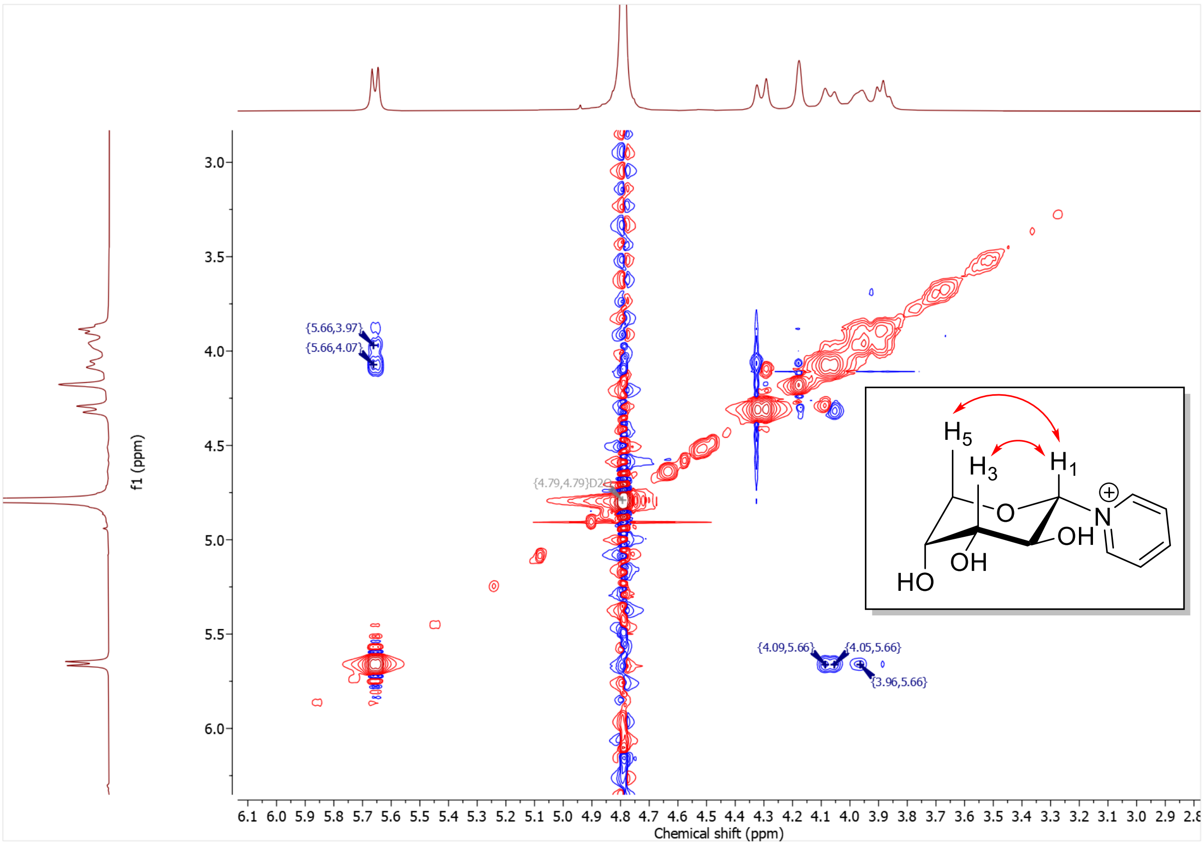

Figure S27: ^{1}H-^{1}H NOESY spectrum of **5b**

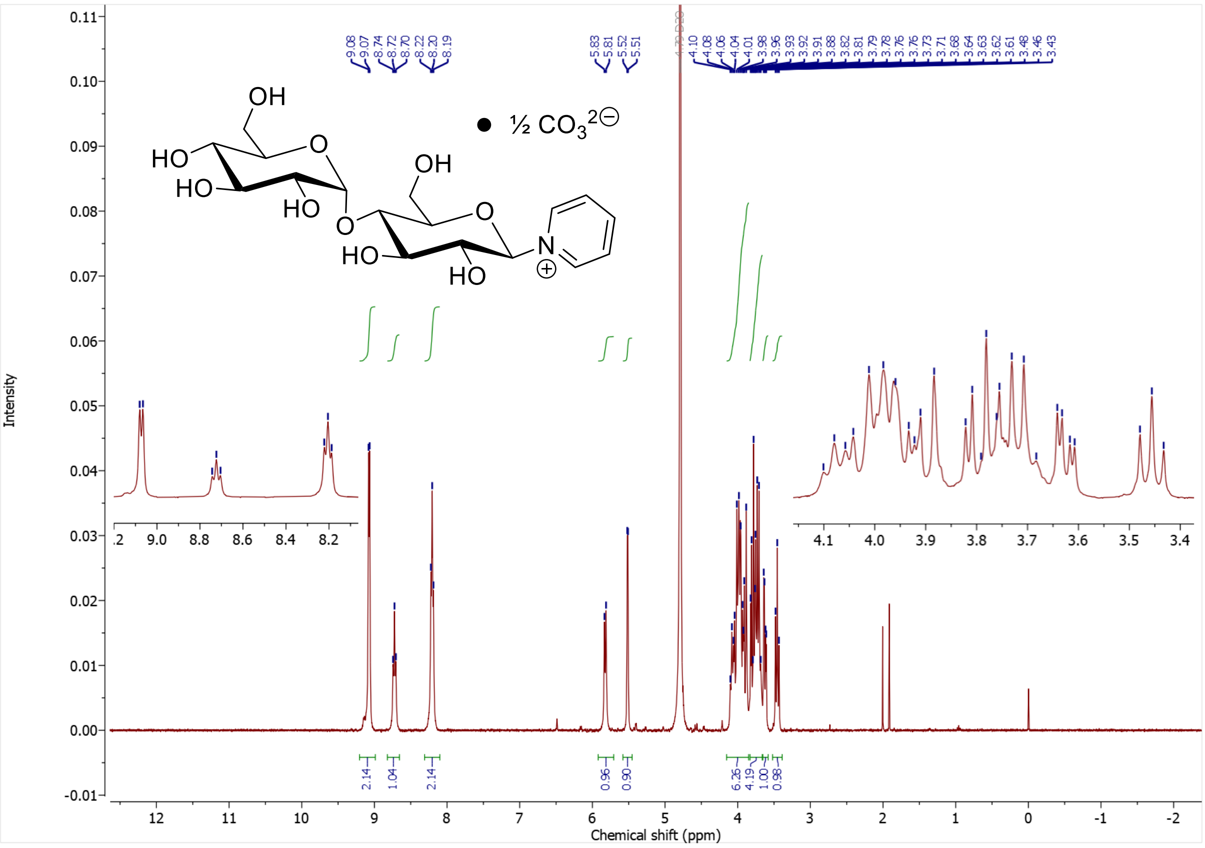

Figure S28: ^{1}H NMR spectrum of **6b**

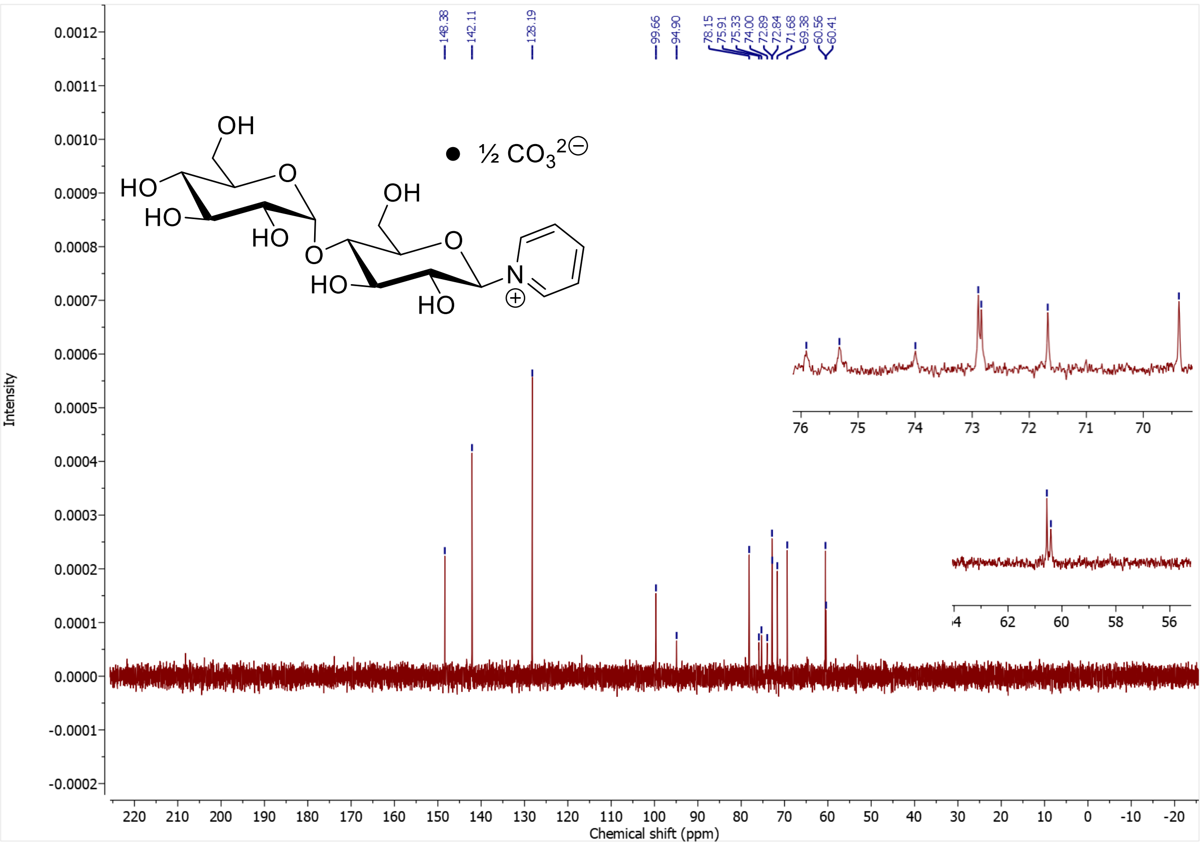

Figure S29: ^{13}C NMR spectrum of **6b**

Classification: In-Confidence

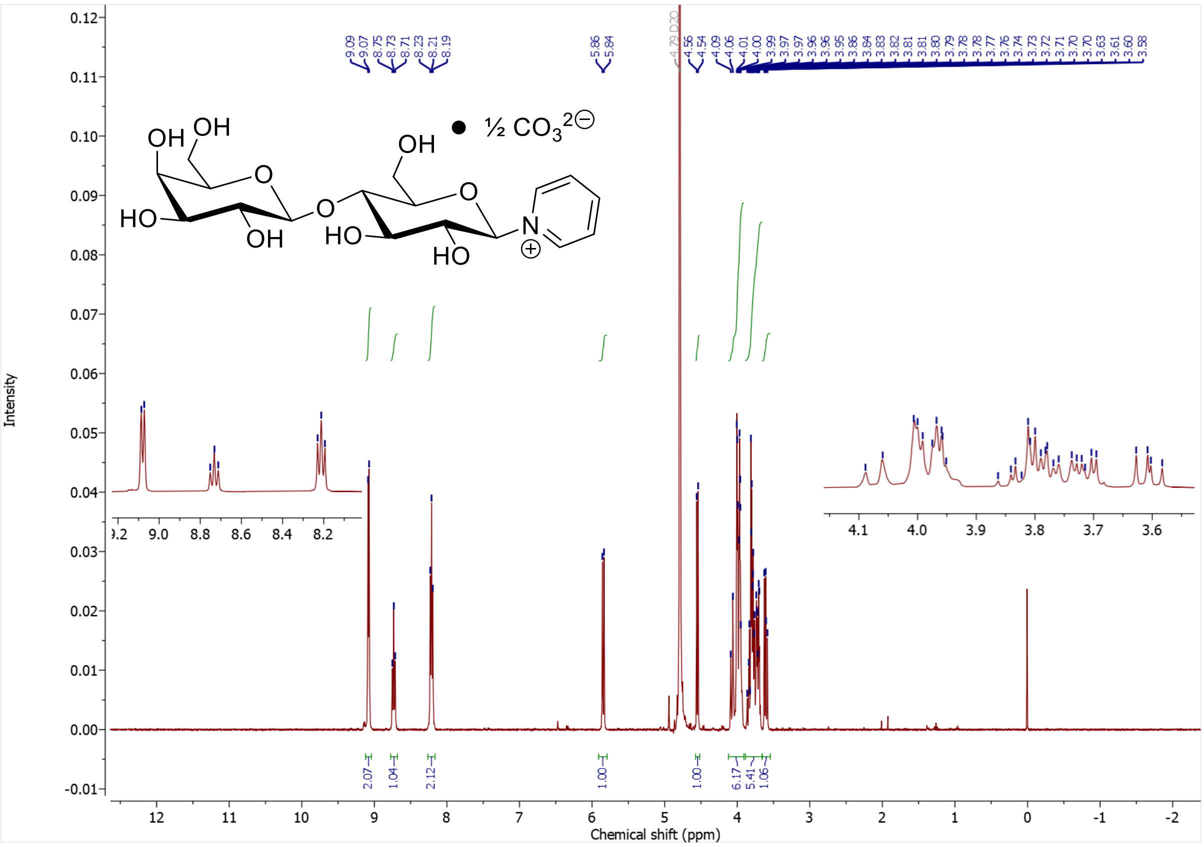

Figure S30: ^{1}H NMR spectrum of **7b**

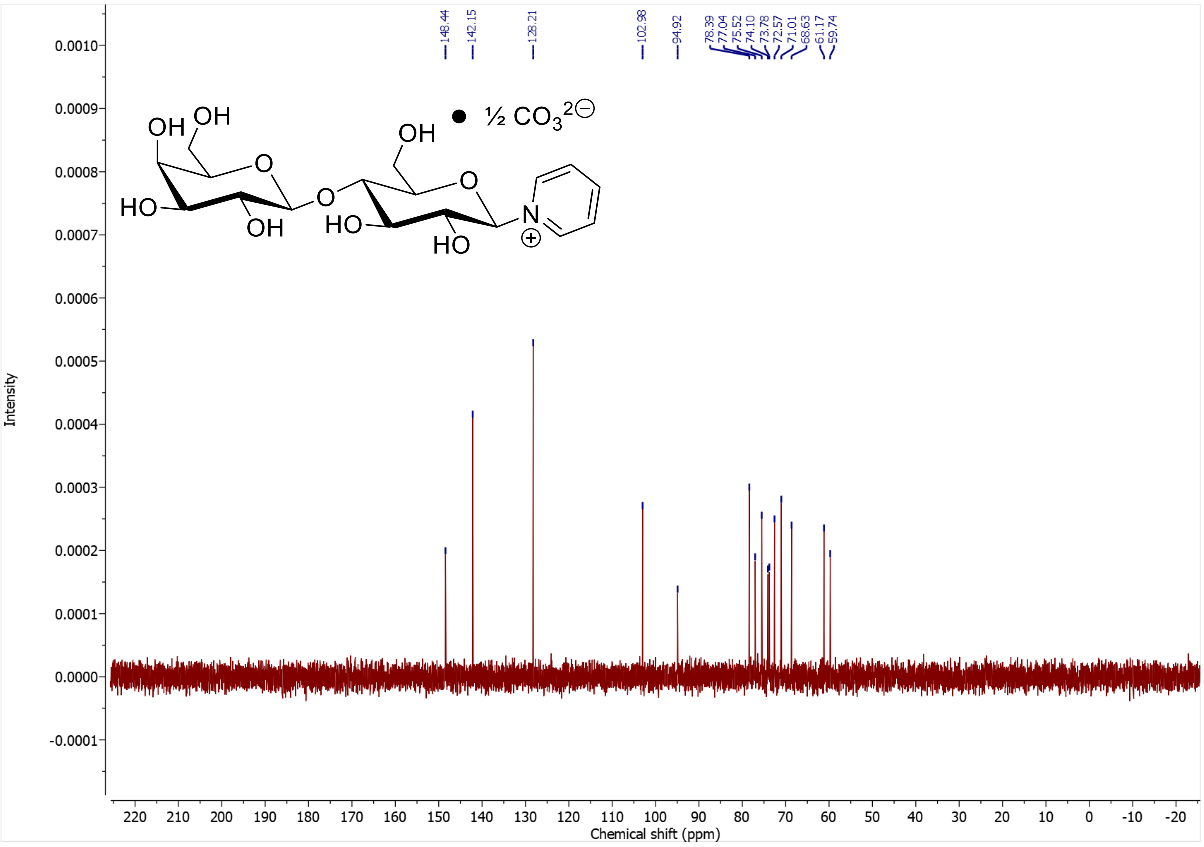

Figure S31: ^{13}C NMR spectrum of **7b**

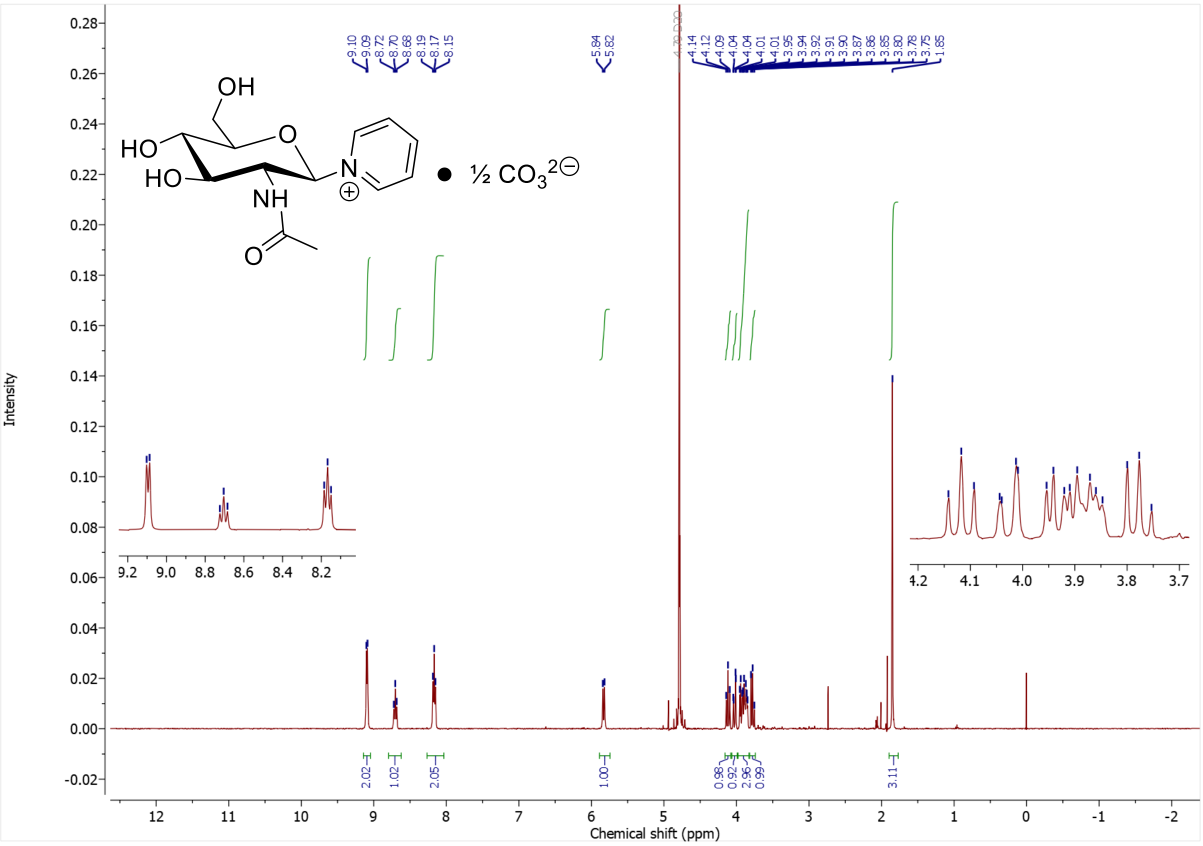

Figure S32: ^{1}H NMR spectrum of **8b**

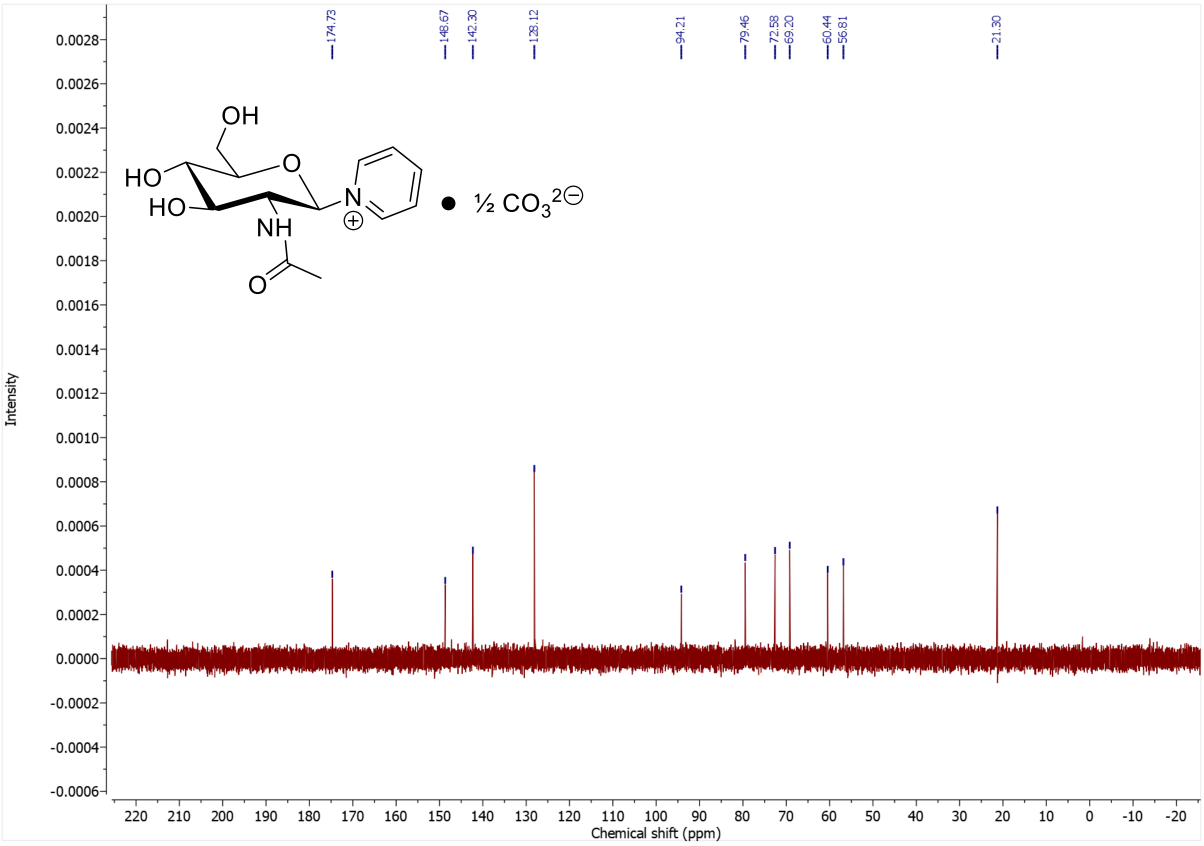

Figure S33: ^{13}C NMR spectrum of **8b**

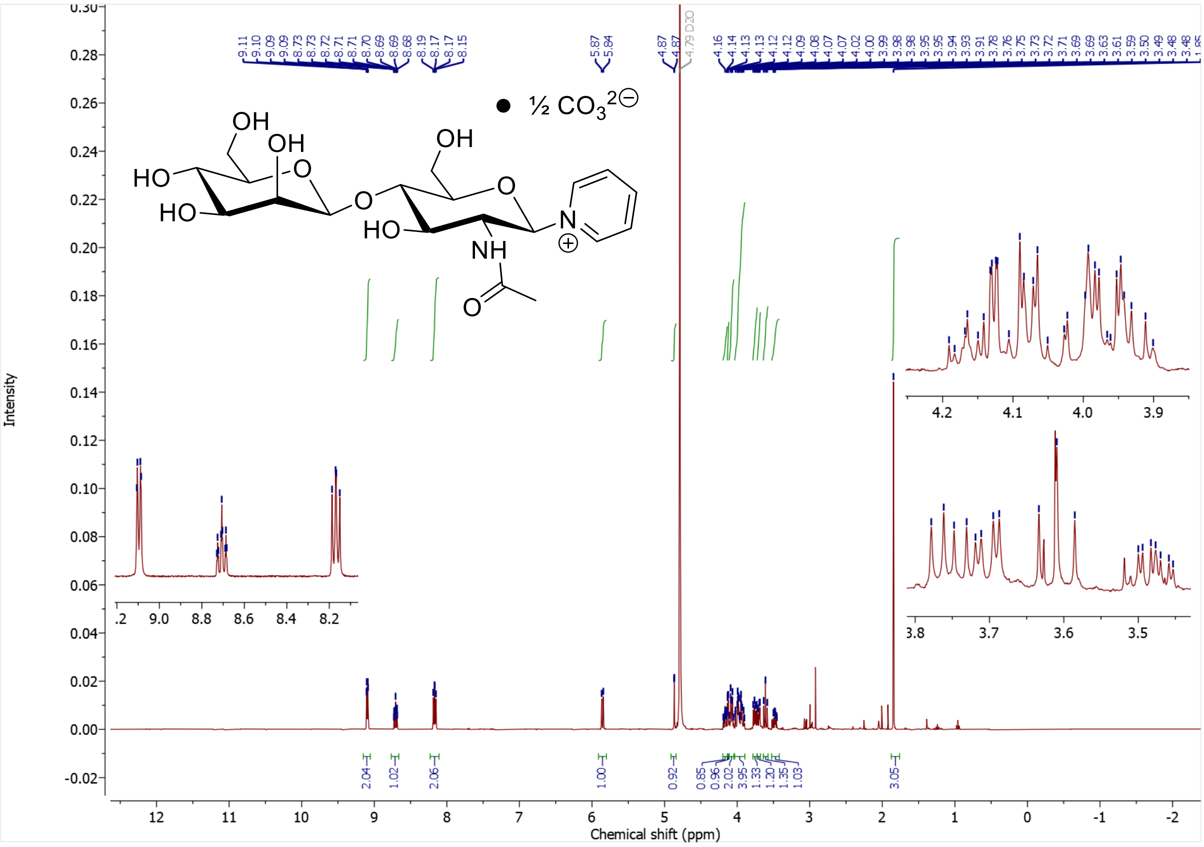

Figure S34: ^{1}H NMR spectrum of **9b**

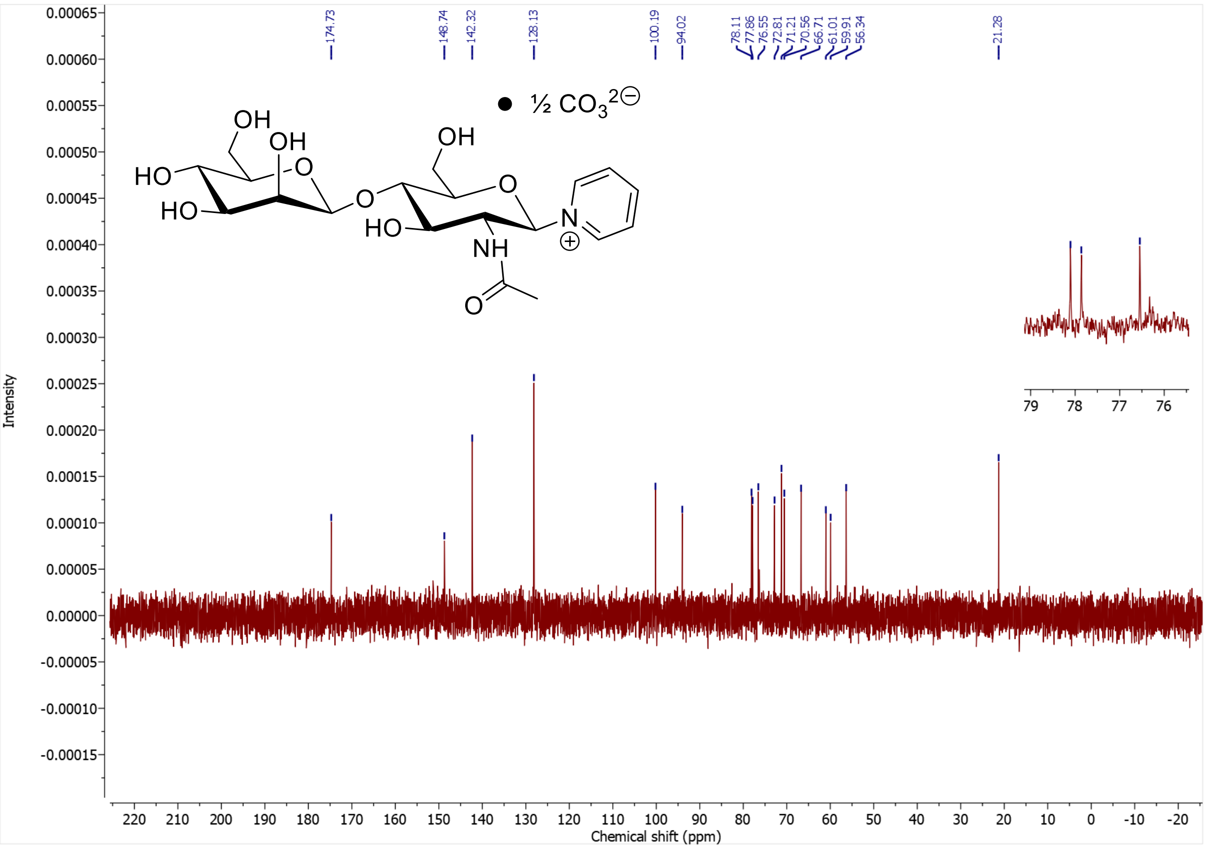

Figure S35: ^{13}C NMR spectrum of **9b**
